# Cucurbit[7]uril Complexation of Near-Infrared Fluorescent Azobenzene-Cyanine Conjugates

**DOI:** 10.3390/molecules27175440

**Published:** 2022-08-25

**Authors:** Sai Shradha Reddy Kommidi, Bradley D. Smith

**Affiliations:** Department of Chemistry and Biochemistry, 251 Nieuwland Science Hall, University of Notre Dame, Notre Dame, IN 46556, USA

**Keywords:** near-infrared fluorescence, cyanine dye aggregation, supramolecular chemistry, azobenzene, cucurbituril

## Abstract

Two new azobenzene heptamethine cyanine conjugates exist as dispersed monomeric molecules in methanol solution and exhibit near-infrared (NIR) cyanine absorption and fluorescence. Both conjugates form non-emissive cyanine H-aggregates in water, but the addition of cucurbit[7]uril (CB7) induces dye deaggregation and a large increase in cyanine NIR fluorescence emission intensity. CB7 encapsulates the protonated azonium tautomer of the 4-(*N*,*N*-dimethylamino)azobenzene component of each azobenzene–cyanine conjugate and produces a distinctive new absorption band at 534 nm. The complex is quite hydrophilic, which suggests that CB7 can be used as a supramolecular additive to solubilize this new family of NIR azobenzene–cyanine conjugates for future biomedical applications. Since many azobenzene compounds are themselves potential drug candidates or theranostic agents, it should be possible to formulate many of them as CB7 inclusion complexes with improved solubility, stability, and pharmaceutical profile.

## 1. Introduction

As part of an ongoing program to invent new classes of near-infrared fluorescence imaging technologies, we are developing supramolecular strategies to manipulate the physical and spectral properties of heptamethine cyanine dyes [1]. These dyes exhibit absorption and fluorescence emission bands within the NIR-I (near-infrared I) window of 700–950 nm for effective fluorescence imaging and sensing of living subjects. Moreover, there is active chemical interest in modifying the dye structures to achieve absorption and emission wavelengths in the NIR-II region of 1000–1700 nm [2]. The extensive π-electron delocalization that is needed to achieve these long wavelengths means the dye molecules are inherently hydrophobic and they have a strong propensity to self-aggregate in water. This hinders the in vivo fluorescence imaging performance of dye-derived molecular probes in several ways. In particular, self-aggregation of the probe molecules can alter the photophysical properties and their pharmacokinetic profiles [3]. In the case of heptamethine cyanine dyes, there is a high likelihood of forming face-to-face H-aggregates, which exhibit blue-shifted absorption bands that are usually not fluorescent [4,5]. One way to diminish dye hydrophobicity is to append charged, polar functional groups to the dyes such as sulfonates [5]. A second approach is to develop supramolecular methods that encapsulate the dyes within discrete water-soluble container molecules such as cyclodextrins or cucurbiturils [6,7].

**Scheme 1:**
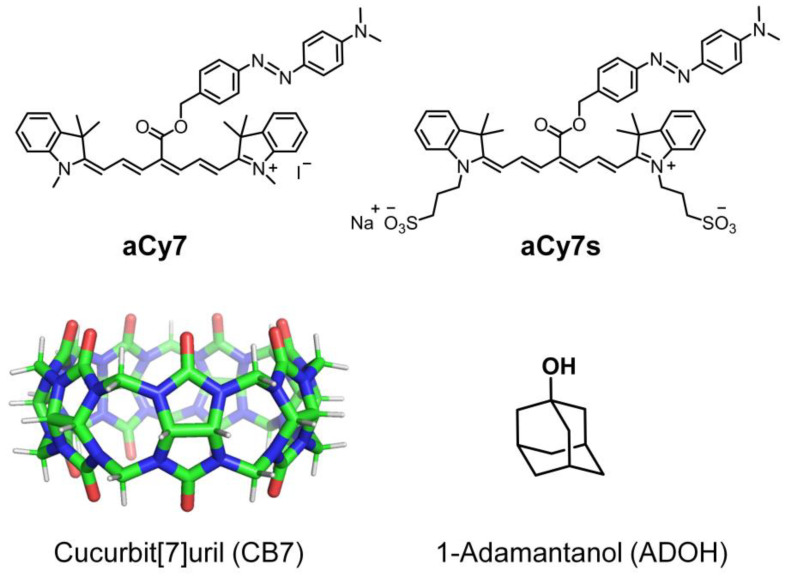
Molecules used in this study.

Within the family of cucurbiturils, CB7 (Scheme 1) is a particularly valuable container molecule because it has high water solubility and the capacity to capture a wide number of guest structures including many drugs and fluorescent dyes [8,9,10]. A large body of work over the last 25 years has shown that CB7 can be exploited for many different supramolecular applications including formulation, delivery, capture, or sensing [11]. In principle, the cavity of CB7 is large enough to accommodate a relatively narrow heptamethine chain, but the sparse literature on cyanine dye complexation by CB7 only contains a few reports describing the unambiguous capture of heptamethine structures that have flat terminal heterocycles such as quinolines [12,13]. Notably, there is no definitive evidence for strong CB7 complexation of heptamethine cyanine dyes that have dimethyl indolinenes as the terminal heterocycles. This is unfortunate because dimethyl indolinenes are by far the most common terminal heterocycles within heptamethine cyanine dyes. A previous study found that the bulky indolinene gem-dimethyl groups at each end of the cyanine structure are large enough to block dye penetration into the CB7 cavity [1], and at the beginning of this study, we experimentally confirmed this fact with a simple set of titration studies that are described in the Supplementary Materials (see Figures S1 and S2).

One pragmatic way to enable the CB7 complexation of a heptamethine cyanine dye is to covalently append a CB7 binding unit to the cyanine dye such as adamantylamine, hexamethylenediamine, or ferrocene [10,14,15,16]. We were attracted to azobenzene as a relatively unexplored CB7 binding unit [17,18,19,20,21] and decided to investigate covalent azobenzene–cyanine conjugates. We reasoned that the conjugates might possess interesting hybrid properties that combine the NIR fluorescence of the cyanine component with the bioresponsiveness of the azobenzene component [22,23,24,25,26]. We also wondered if the inherent hydrophobicity of azobenzene–cyanine conjugates could be overcome by CB7 complexation.

Here, we report our first set of results; that is, we describe the synthesis and properties of two novel azobenzene–cyanine conjugates, namely **aCy7** and **aCy7s** (Scheme 1).

We show that they readily form nonfluorescent H-aggregates in water, and that dye self-aggregation is reversed upon the addition of CB7, which captures the azobenzene component of the conjugate and switches on NIR fluorescence emission by the cyanine component. Moreover, CB7 encapsulates the azobenzene component as a protonated azonium structure, which is distinctly different from the azobenzene capture mechanism exhibited by cyclodextrins and albumin protein. The conclusion section describes possible future applications of this unique dye capture process in NIR fluorescence imaging, diagnostics, and drug formulation.

## 2. Results and Discussion

### 2.1. Synthesis

**Scheme 2:**
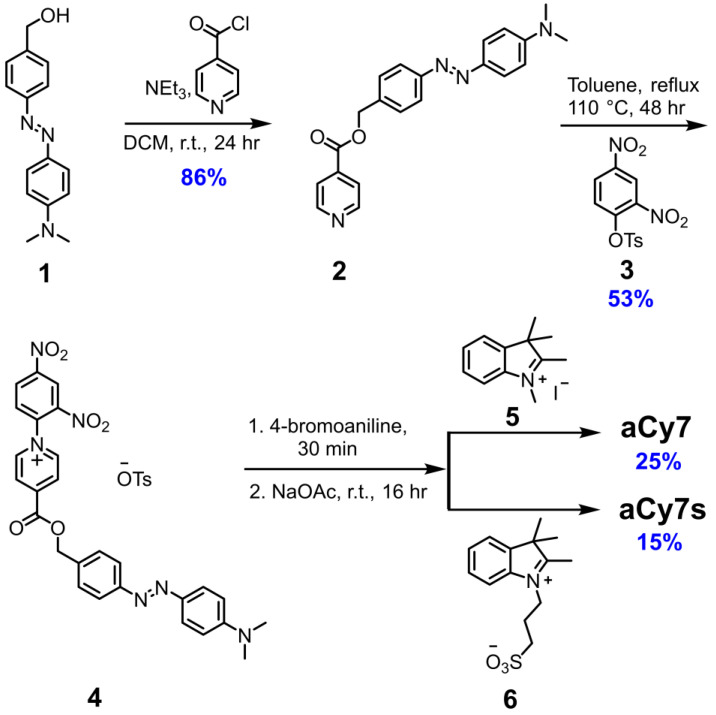
Synthesis of **aCy7** and **aCy7s**.

The known compound **1** was prepared [27] and reacted with isonicotinic acid chloride to obtain the corresponding isonicotinate ester **2**, which was refluxed with 2,4-dinitrophenyl p-toluenesulfonate **3** in toluene to avail the pyridinium Zincke salt **4,** as shown in Scheme 2. Separate reactions of **4** with indolinium salts **5** or **6** produced the azobenzene–cyanine conjugates **aCy7** or **aCy7s**, respectively [28]. The cationic **aCy7** was purified using column chromatography (SiO_2_, 0–5% MeOH in DCM), whereas reverse-phase column chromatography (C18, 50–90% MeOH containing 0.5% TFA in distilled water) was employed to purify anionic **aCy7s**. The two azobenzene–cyanine conjugates were characterized by NMR, mass spectrometry, absorption, and fluorescence spectroscopy.

### 2.2. Spectral Properties of Dyes and Solvatochromism

**Figure 1:**
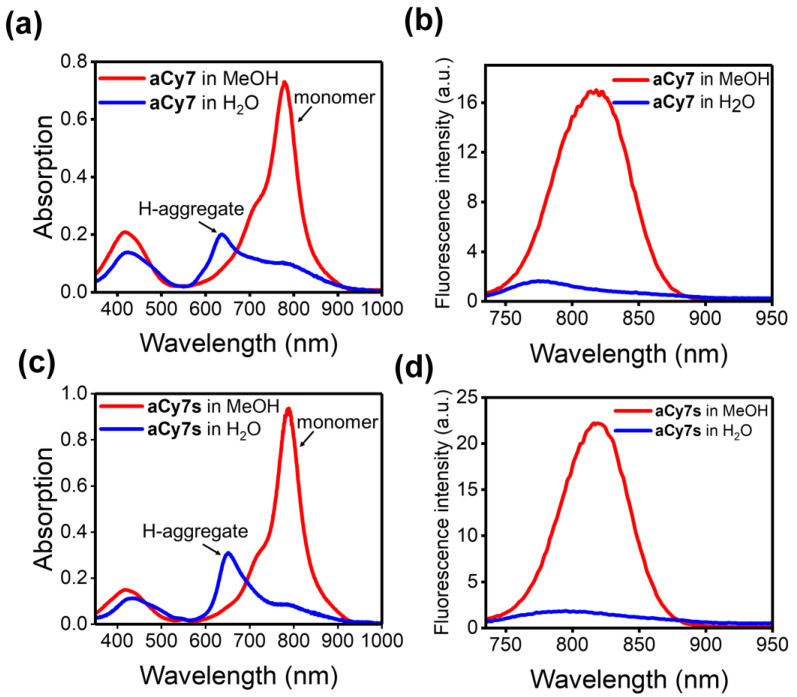
Absorption (**a**,**c**) and emission (**b**,**d**) spectra of **aCy7** (5 µM) and **aCy7s** (5 µM), respectively, in methanol (red) and water (blue) at room temperature. λ_ex_ = 720 nm; slit width = 5 nm.

In methanol, the azobenzene–cyanine conjugates **aCy7** and **aCy7s** exhibited relatively sharp cyanine absorption bands with maxima wavelengths of 780 and 786 nm, respectively (Figure 1a,c and Table 1). Both dyes emit around 820 nm (Figure 1b,d), and the Stokes shift of ~35 nm is somewhat larger than many heptamethine cyanine dyes. The quantum yields for the cyanine fluorescence, *Φ_f_* = 0.36% for **aCy7s** and *Φ_f_* = 0.30% for **aCy7,** match the low values measured for similar-literature heptamethine cyanine dyes with 4′-ester substituents [29]. In other words, the appended 4-(*N*,*N*-dimethylamino)azobenzene component does not quench the near-infrared fluorescence of the heptamethine cyanine component. The absorption band at 434 nm is assigned to the 4-(*N*,*N*-dimethylamino)azobenzene chromophore as the thermodynamically preferred trans azobenzene isomer. It is known that the π→π∗ and n→π∗ transitions for 4-(*N*,*N*-dimethylamino)azobenzene derivatives closely overlap, which makes it difficult to produce or observe light-induced interconversion of trans and cis isomers of the azobenzene component in **aCy7** and **aCy7s** [30].

In water, the absorption maxima of the respective azobenzene component within **aCy7** and **aCy7s** was slightly red-shifted compared with methanol, whereas the cyanine component was strongly blue-shifted and appeared at 634 or 654 nm, respectively, with relatively low peak intensities (Figure 1a,c). The large cyanine blue shifts are hallmarks of cyanine dye self-association and formation of face-to-face H-aggregates [3,4,5]. Moreover, the cyanine fluorescence emission for each dye was strongly quenched in water compared with methanol (Figure 1b,d), which is another hallmark of cyanine H-aggregation. Further characterization of the H-aggregates was gained by analyzing separate samples of **aCy7** and **aCy7s** in an aqueous solution using dynamic light scattering (DLS) (Figure S5) and determining the intensity weighted mean hydrodynamic size of the aggregates (Z_avg_). The aggregate size for **aCy7** (Z_avg_ = 1062 nm) was observed to be larger than the aggregate size for **aCy7s** (Z_avg_ = 825 nm), and the difference is attributed to the presence of two anionic sulfonate groups in **aCy7s** that enhance the dye’s hydrophilicity.

An initial set of dye solvatochromism experiments acquired absorption spectra for **aCy7** and **aCy7s** in different water–methanol mixtures and observed the expected trend of decreased dye aggregation with increased mole fraction of methanol (Figure S3). A second set of solvatochromism experiments compared absorption spectra for **aCy7** and **aCy7s** in four different solvents: water, methanol, DMSO, and DCM (Figure S4). In the case of cationic and lipophilic **aCy7**, the spectra indicated decreased dye aggregation in the less polar solvent (Figure S4a). In the case of sulfonated **aCy7s**, there was less aggregation in methanol or DMSO compared with water, but in the least polar organic solvent, DCM, there was a strong aggregation band at 672 nm (Figure S4b). Moreover, the absorption spectra in DCM–methanol mixtures showed decreased dye aggregation with increased mole fraction of methanol (Figure S4c). Presumably, the face-to-face H-aggregate structure for **aCy7s** in water is different from the H-aggregate structure in DCM. In water, the sulfonates on **aCy7s** are likely to be exposed to the solvent, but in DCM, they are likely to be buried and protected from the solvent [32,33].

### 2.3. Binding Studies with CB7

**Figure 2:**
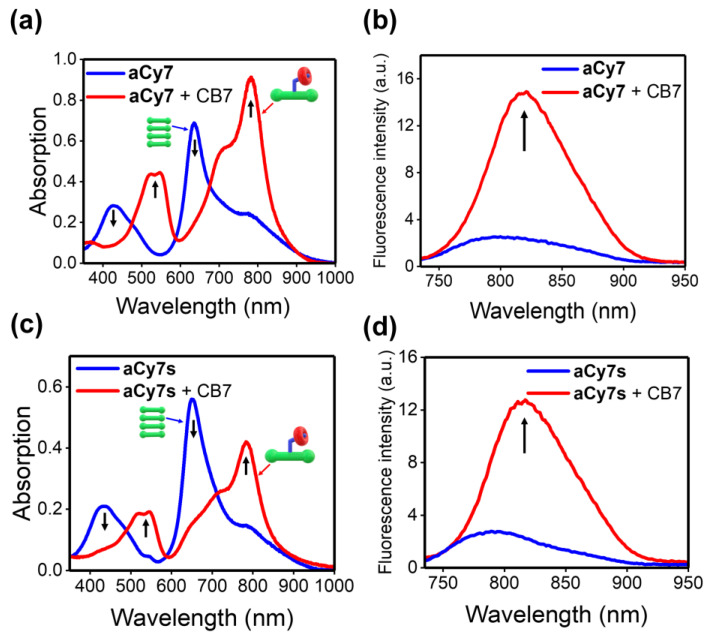
Absorption (**left**) and emission (**right**) spectra (10 µM, blue) of **aCy7** (**a**,**b**) and **aCy7s** (**c**,**d**) and their CB7 complexes (red, 30 molar equivalents of CB7) in distilled water at pH 5.70 at room temperature. λ_ex_ = 720 nm; slit width = 5 nm.

**Figure 3:**
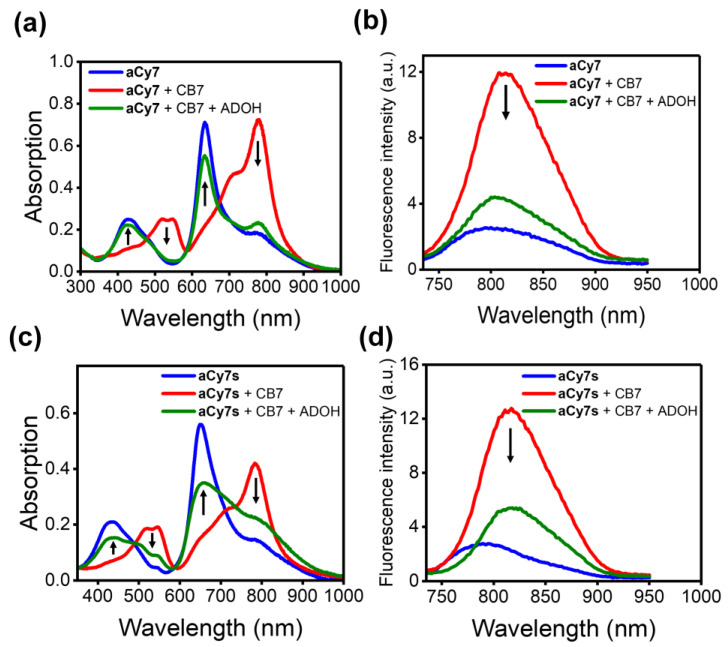
(**a**,**b**) Absorption (**left**) and emission (**right**) spectra of the complex **CB7@aCy7** before (red, 10 molar equivalents of CB7) and after addition of 1-adamantanol (ADOH, green, 10 molar equivalents) in distilled water, pH 5.70, at room temperature. (**c**,**d**) Absorption (**left**) and emission (**right**) spectra of complex **CB7@aCy7s** before (red, 30 molar equivalents of CB7) and after addition of ADOH (green, 10 molar equivalents) in water, pH 5.70, at room temperature. λ_ex_ = 720 nm; slit width = 5 nm.

The addition of excess CB7 to separate samples of **aCy7** (or **aCy7s**) in water produced major changes in the absorption and fluorescence spectra. The absorption spectra showed conversion of the azobenzene peak at 434 nm into a peak at 534 nm (Figure 2a,c) and conversion of the cyanine H-aggregate peak at 635 nm (or 654 nm) into a monomer cyanine absorption peak at 786 nm. The combined effect of these spectral conversions is an observable change in sample color from green to pink (Figure S10). In addition, the NIR fluorescence emission intensity upon excitation of the cyanine peak increased by 84% and 78% for **aCy7** and **aCy7s,** respectively (Figure 2b,d), and closely matched the NIR fluorescence profiles observed in methanol. In both cases, the absorption and fluorescence changes were reversed by displacement of the **aCy7** or **aCy7s** dye from the CB7 host. This was achieved by adding to the CB7 + dye mixture an aliquot of 1-adamantanol (Figure 3), a high-affinity guest for CB7 (*K_a_* ∼10^10^ M^−1^) [34,35]. The dyes and CB7 complexes in water could not be studied by ^1^H NMR due to extensive self-aggregation at the relatively high concentrations needed for NMR analysis. However, mass spectra of aqueous samples containing a binary mixture of CB7 + **aCy7** or CB7 + **aCy7s** produced peaks corresponding to CB7–dye complexes with 1:1 stoichiometry (Figures S6 and S7).

**Figure 4:**
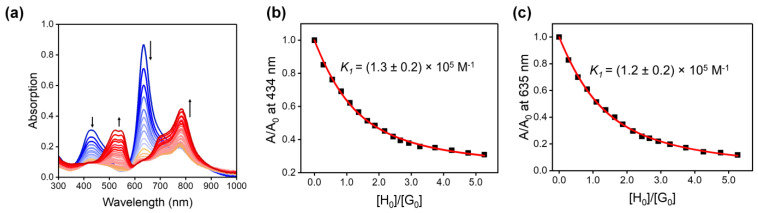
(**a**) Absorption spectra of 10 µM **aCy7** with increasing amounts of CB7 (0.0 eq–40.0 eq) in water, pH 5.70, at room temperature. Each absorption spectrum was acquired after letting the solution equilibrate for 5 min. The blue lines indicate decreasing spectral intensity and the red lines indicate increasing spectral intensity. (**b**,**c**) Representative titration isotherms showing absorption for **aCy7** at 434 and 635 nm with increasing equivalents of CB7 in water, along with fitting to a 1:1 binding model (red line).

Shown in Figure 4a are the results of a titration experiment that added CB7 to a solution of **aCy7** and tracked changes in the absorption spectra. Upon the incremental addition of CB7, the absorbance peaks of the azobenzene component at 434 nm and the cyanine component at 635 nm decreased and were replaced by the corresponding peaks at 534 and 786 nm, respectively. The titration did not produce a well-defined isosbestic point, suggesting multiple equilibria. Nonetheless, we could determine an approximate association constant for the first equilibrium step (*K_1_*) that forms **CB7@aCy7** by fitting the isotherms in Figure 4b,c to a 1:1 binding model. The two independent values of *K_1_* were (1.3 ± 0.2)\1 × \210^5^ M^−1^ from the decrease in azobenzene absorbance at 434 nm and (1.2 ± 0.2)\1 × \210^5^ M^−1^ from the decrease in cyanine absorbance at 635 nm. CB7 titration studies with the anionic sulfonate version **aCy7s** were also performed, and similar changes in absorption spectra were obtained (Figure S8); however, the isotherm did not match with a 1:1 binding model [36]. Qualitatively, it was apparent that CB7 has greater affinity for cationic **aCy7** than for anionic **aCy7s** (compare the titration data in Figure 4 and Figure S8), which is consistent with the preference of CB7 for cationic molecular guests [37].

**Figure 5:**
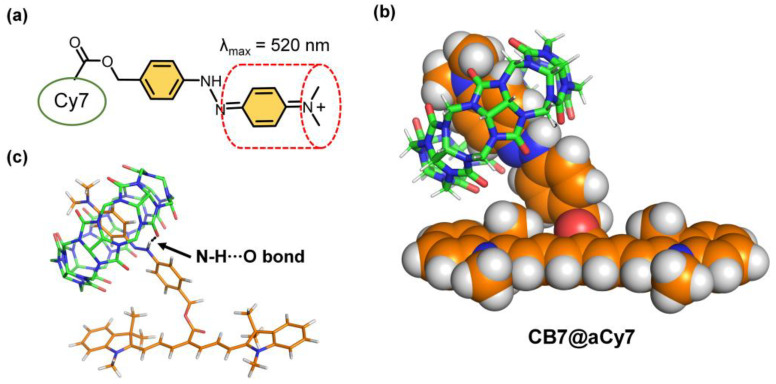
(**a**) Chemical structure of protonated 4-(*N*,*N*-dimethylamino)azobenzene (azonium tautomer) encapsulated by CB7. (**b**) DFT-calculated structure of **CB7@aCy7** complex in water at room temperature. (**c**) Wireframe version of calculated **CB7@aCy7** complex depicting N-H•••O hydrogen bond between portals of CB7 and protonated azonium tautomer (hydrogen in CB7 has been omitted for clarity reasons).

Two sets of independent experimental evidence indicate that CB7 much more strongly encapsulated the azobenzene component of the azobenzene–cyanine conjugates than the cyanine component. As stated in the introduction section, a titration experiment that added CB7 to a control heptamethine cyanine dye bearing terminal dimethyl indolinene units but no appended azobenzene component (see Figures S1 and S2) produced minor changes in absorption spectra and a low *K_a_* value of (5.2 ± 0.7)\1 × \210^2^ M^−1^. Logically, the much higher *K_1_* for the formation of **CB7@aCy7** must be due to CB7 encapsulation of its azobenzene component. CB7 encapsulation of the azobenzene component also explains the conversion of the azobenzene absorption peak at 434 nm into a peak at 534 nm (Figure 2a,c). The same spectral change was recently reported by He and coworkers in a study that used CB7 to encapsulate methyl orange, a structurally related 4-(*N*,*N*-dimethylamino)azobenzene dye [21]. Moreover, an X-ray crystal structure of the CB7–methyl orange complex showed that the CB7 encapsulated the protonated form of the 4-(*N*,*N*-dimethylamino)azobenzene chromophore with the proton located on the azo group (azonium tautomer) and hydrogen bonded to the surrounding CB7. With the coordinates of this crystal structure as a starting point, we used the density functional theory to compute the local minimized energy structure for **CB7@aCy7** (1:1 complex). As shown in Figure 5, CB7 is positioned over the protonated azonium group of the 4-(*N*,*N*-dimethylamino)azobenzene component of **aCy7**. The protonated form of a 4-(*N*,*N*-dimethylamino)azobenzene chromophore has a p*K*_a_∼3.2, which is lower than the pH of the aqueous titration solvent (the final pH at the end of the titration was 4.80); nonetheless, the azonium cation is stabilized within CB7 by an internal hydrogen bond with CB7 as indicated in Figure 5c. This picture is consistent with the known propensity of CB7 to bind a molecular guest as its cationic conjugate acid [38,39]. Thus, the absorption band at 430 nm corresponds to the neutral azo form of the 4-(*N*,*N*-dimethylamino)azobenzene component when it is free in water, and the peak at 534 nm corresponds to its protonated form (azonium tautomer) when encapsulated by CB7 [40,41,42].

**Scheme 3:**
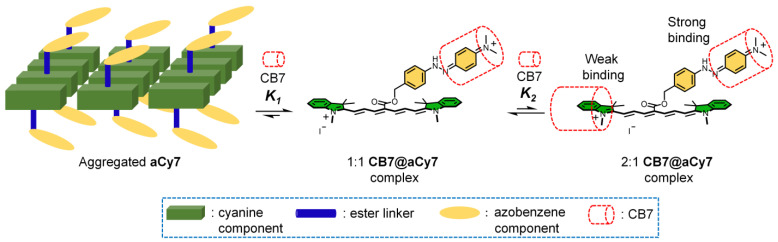
Supramolecular model for **aCy7** self-aggregation and encapsulation by CB7 in water.

The complete supramolecular association process is summarized in Scheme 3, which depicts **aCy7** as the azobenzene–cyanine dye conjugate. In water, **aCy7** self-aggregates, which produces a blue-shifted cyanine absorption band (cyanine H-aggregate) that is nonfluorescent. At the same time, there is a smaller red shift in absorption for the azobenzene component, indicating an end-to-end alignment of azobenzenes within the aggregate (azobenzene J-aggregate) [43,44]. When CB7 is added to the sample, it encapsulates the protonated azobenzene component (azonium tautomer), which converts the azobenzene absorption band at 430 nm into a peak at 534 nm and converts the cyanine H-aggregate peak at 635 nm into a monomer absorption peak at 786 nm that emits NIR fluorescence. When a large excess CB7 is added to the sample, there is additional weak binding of CB7 to one of the terminal dimethyl indolinene units on the cyanine component (*K_2_*), but this has only a minor effect on the dye spectral properties.

It is worth noting that when the titrations of **aCy7** or **aCy7s** with CB7 were repeated in phosphate-buffered saline (150 mM NaCl, 10 mM phosphate, pH 7.4), there was very little change in dye spectral properties indicating negligible dye encapsulation by CB7. This was expected for two reasons: (i) sodium cations strongly associate with the polar carbonyls at each portal of CB7 and inhibit dye encapsulation [45,46], and (ii) complexation-induced protonation of the 4-(*N*,*N*-dimethylamino)azobenzene component of the dye conjugates is inhibited by the higher solution pH of 7.4.

### 2.4. Binding Studies with Cyclodextrins and Albumin Protein

Cyclodextrins (CDs) are another class of water-soluble container molecules, and they are known to encapsulate azobenzene derivatives [47,48,49]. We evaluated the capabilities of five different CDs (α-CD, β-CD, γ-CD, 2-hydroxypropyl β-CD, and methyl β-CD; see Supplementary Materials for chemical structures) to bind **aCy7** or **aCy7s** and deaggregate the dyes. The absorption and fluorescence data in Figures S11–S20 show that the presence of α-CD, β-CD, γ-CD, or 2-hydroxypropyl β-CD induces small or modest changes in spectral profiles. Only in the case of methyl β-CD was there a large change in the absorption and fluorescence profiles of the cyanine component within **aCy7** or **aCy7s**. More specifically, there was a significant decrease in the cyanine H-aggregate band and concomitant increase in the cyanine monomer peak, indicating supramolecular encapsulation of the azobenzene–cyanine conjugate by methyl β-CD. Notably, there was a negligible change in the absorption peak at 430 nm for the 4-(*N*,*N*-dimethylamino)azobenzene component of the dye conjugates indicating no formation of the azonium tautomer band at 530 nm that is seen when **aCy7** or **aCy7s** is captured by CB7.

The same trend was observed when separate samples of **aCy7** or **aCy7s** were treated with bovine serum albumin (BSA), a common blood protein that is known to bind cyanine dyes and disrupt cyanine dye H-aggregates [50,51,52]. As shown in Figures S21 and S22, the presence of BSA converted the cyanine H-aggregate band into a cyanine monomer peak, indicating the association of the azobenzene–cyanine conjugate with the BSA. Moreover, the effect was larger for anionic **aCy7s**, which is consistent with the known preference of BSA to strongly bind anionic amphiphiles [53]. However, there was no change in the absorption peak at 430 nm for the 4-(*N*,*N*-dimethylamino)azobenzene component of the dye conjugates indicating that it remains in its nonprotonated azo form.

### 2.5. Feasibility of Azobenzene–Cyanine Conjugates for Use in Biomedical Systems

To provide a preliminary assessment of feasibility for future biomedical studies using azobenzene–cyanine conjugates, we conducted three sets of independent experiments using cationic **aCy7**, which had the higher affinity for CB7.

**Figure 6:**
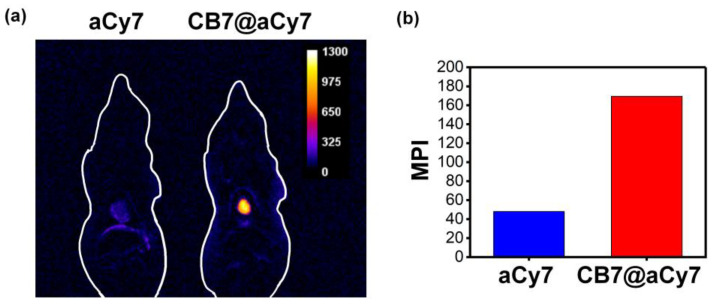
(**a**,**b**) NIR fluorescence images (λ_ex_ = 745 nm, λ_em_ = 850 nm) and resultant mean pixel intensity graphs of two mouse phantoms with heart portals, containing 10 µM **aCy7** (left) and **aCy7** CB7 complex (right, **aCy7** + 30 molar eq. of CB7) in distilled water.

From the perspective of NIR fluorescence imaging of living subjects, we assessed the benefit of CB7 as a formulation additive that deaggregates **aCy7** in water. Using a commercial small-animal in vivo imaging station, we mimicked a typical imaging scenario by imaging mouse phantoms that contained a fillable portal corresponding to the heart. Shown in Figure 6a is a comparison of NIR fluorescence images for two identical mouse phantoms in the same imaging station. The heart portal of each phantom was filled with an aqueous solution of either the **aCy7** or **CB7@aCy7** complex. Comparison of the NIR fluorescence images in Figure 6a shows that the signal from the portal containing the **CB7@aCy7** complex is much brighter because the **aCy7** dye is not self-aggregated. Quantification of the images was achieved by measuring the mean pixel intensity for each image, and the bar graph in Figure 6b reflects a fourfold increase in image brightness—a major enhancement of in vivo fluorescence imaging performance.

**Figure 7:**
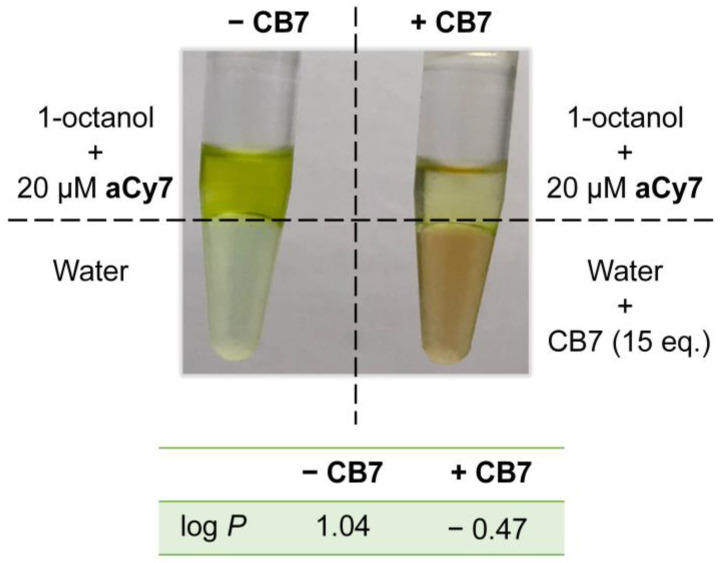
Photographs of samples and log *P* values for 20 µM **aCy7** in the presence and absence of CB7 in the water layer (15 molar equivalents of CB7).

Not only does self-aggregation of **aCy7** lead to the quenching of cyanine fluorescence but also the high lipophilicity of the aggregate is likely to alter the in vivo pharmacokinetic profile and produce off-target association with undesired biological surfaces [3]. Since CB7 is quite water-soluble, we measured its effect on **aCy7** partitioning between water and organic 1-octanol. As shown by the photograph in Figure 7, lipophilic **aCy7** prefers the 1-octanol phase, and a log *P* of 1.04 ± 0.02 was determined (*P* is the partition coefficient). When CB7 was included in the two-phase mixture, there was an obvious transfer of **aCy7** to the water layer resulting in a negative in the log *P* value of —0.47 ± 0.01. In other words, the **CB7@aCy7** complex is substantially more hydrophilic than free **aCy7**. Thus, formulation of azobenzene–cyanine conjugates as their CB7 inclusion complexes is a promising future strategy to increase conjugate water solubility and fine-tune the pharmacokinetics [54].

The last set of feasibility experiments assessed the likely biocompatibility of **aCy7**, CB7, and **CB7@aCy7** by measuring their effects on cell metabolic activity using a standard MTT assay. Treatment of MDA-MB-231 cells (breast adenocarcinoma) for 72 h with concentrations of **aCy7**, CB7, or **CB7@aCy7** ranging from 0 to 300 µM produced very little change in the cell metabolic activity (Figure S23) suggesting that these compounds have high biocompatibility.

## 3. Materials and Methods

### 3.1. General Methods

Reagents and solvents were purchased from Sigma-Aldrich (Burlington, Massachusetts, USA), VWR (Radnor, PA, USA), Oakwood (Estill, SC, USA), Alfa Aesar (WardHill, MA, USA), Thermo Fisher (Waltham, MA, USA) and used without further purification unless stated otherwise. Analyte solutions were prepared in HPLC-grade methanol (Sigma-Aldrich, Burlington, MA, USA), HPLC-grade dimethyl sulfoxide (Alfa Aesar), HPLC-grade DCM (Alfa Aesar, WardHill, Massachusetts, USA), or phosphate-buffered saline 1×, pH = 7.4 (Thermo Fisher, Waltham, MA, USA). All absorption and fluorescence spectra were collected using quartz cuvettes (1 mL, 1 cm path length; for emission and excitation spectra, slit width = 5 nm, unless stated otherwise). Flash column chromatography was performed using Biotage SNAP Ultra columns. ^1^H and ^13^C NMR spectra were recorded on a Bruker 400 or Bruker 500 NMR spectrometer. Chemical shifts are presented in ppm and referenced by residual solvent peak. High-resolution mass spectrometry (HRMS) was performed using a time-of-flight (TOF) analyzer with electrospray ionization (ESI). Absorption spectra were recorded on an Evolution 201 UV–vis spectrometer with Thermo Insight software. Fluorescence spectra were collected on a Horiba Fluoromax4 fluorometer or Fluoromax Plus fluorometer with an InGaAs detector and FluoroEssence software. Unless stated otherwise, all aqueous solutions used distilled water with pH ~ 5.70 due to the absorption of carbon dioxide from the air.

### 3.2. Relative Fluorescence Quantum Yield Measurements

The concentrations of **aCy7** and **aCy7s** were adjusted to the absorption value of 0.075 at 650 nm. The fluorescence spectrum of each solution was obtained with excitation at 650 nm, with a slit width of 5 nm, and the integrated area was used in the quantum yield calculation by the following equation:

$$\Phi_{sample}=\Phi_{ref}\times \frac{\eta_{sample}^{2}\;I_{sample}\;A_{sample}}{\eta_{ref}^{2}\;I_{ref}\;A_{ref}}$$

where *η* is the refractive index of the solvent (*η_methanol_* = 1.328) and is the same for both sample and reference compounds, *I* is the integrated fluorescence intensity, and *A* is the absorbance at a chosen wavelength [55,56]. Ref is the dye **1k** from [31], which acted as a relative standard (*Φ_ref_* = 13.2% in methanol). The estimated error for this method is ±10%.

### 3.3. Association Constant Determinations

Absorption titration experiments were carried out at room temperature using quartz cuvettes (1 mL, 1 cm path length). A solution of 10 μM **aCy7** in distilled water was placed in a 1 mL quartz cuvette and titrated with aliquots from a CB7 stock solution (1.0 mM CB7 and 10 μM dye in distilled water). Following each addition of the host, the solution was allowed to equilibrate for 5 min, and then an absorption spectrum was acquired. At the start of the titration, the pH of the dye solution was ~5.70, and it was 4.80 after all aliquots of CB7 were added. Association constants were determined by plotting the change in the absorbance intensity versus molar equivalents and fitting the curve to a 1:1 binding model using Origin 8.6 software (see Section S5 of the Supplementary Materials). The error is the standard deviation for three measurements. The same *K_1_* values were obtained by fitting the data using Bindfit software [57].

### 3.4. Molecular Modeling

The input structure of the **CB7@aCy7** complex was created by hand using X-ray structure coordinates for the 4-(*N*,*N*-dimethylamino)azobenzene component as its azonium tautomer [21]. The molecular geometry was optimized using MMFF94 force field in Avogadro (1.2.0) [58]. The single-point energy and dipole moment for the complex were calculated using Gaussian16/C.01 with the CAM-B3LYP/6-31G** functional and basis set in water (CPCM model) [31,59,60].

### 3.5. Mouse Phantom Imaging

The fillable mouse phantoms (purchased from Bioemtech) are precise physical models of a 28 g mouse extracted from anatomical and cryosection data and fabricated using a soft-tissue-mimicking material. The major organs are fillable ports, and the fillable port corresponding to the heart was used for imaging within a commercial in vivo imaging station (Spectral AMI HT). To mimic a typical **aCy7** in vivo imaging scenario, the heart port of each phantom mouse was filled with distilled water and an aliquot from a 1.0 mM stock solution of either **aCy7** or **CB7@aCy7** (the final dye concentration was 10 μM, and 30 molar equivalents of CB7 was used to make sure a full complexation was achieved). NIR fluorescence images were acquired, and the mean pixel intensities of the heart regions were determined using ImageJ software as previously described [5].

### 3.6. Log P Determination

Distilled water (990 µL) and 1-octanol (990 µL) were suspended in two separate 1.5 mL Eppendorf tubes. **aCy7** (20 µM) was added to the 1-octanol layer, and the aqueous layer was mixed with water (sample A) or an aliquot of CB7 (300 µM) (sample B), with similar final volumes of aqueous and organic layers. The samples were centrifuged at 1000 rcf for 5 min and then allowed to partition for 30 min. Aliquots (200 µL) from organic and aqueous layers of both tubes were pipetted into a 96-well plate, and the absorbance was measured at 780 nm using a plate reader. The log *P* in the presence and absence of CB7 was calculated by the following equation: log(A_780_ of 1-octanol layer/A_780_ of water layer).

### 3.7. Cell Metabolic Activity Assay

Metabolic activity assays were performed using human breast adenocarcinoma (MDA-MB-231, ATCC HTB-26) treated with free **aCy7**, free CB7, or CB7@**aCy7** (1:1). Cells were cultured in Dulbecco’s Modified Eagle’s Medium (DMEM) growth medium supplemented with 10% (*v*/*v*) fetal bovine serum (FBS), 1% (*v*/*v*) 200 mM L-glutamine, and (100 IU mL^−1^–100 μg mL^−1^) penicillin–streptomycin. Cells were maintained in a humidified 5% CO_2_ incubator at 37 °C and used at a confluence of around 80%. A total of 5 × 10^3^ cells per well were seeded in 96-well plates at 37 °C and 5% CO_2_. Stock solutions (1 mM) of **aCy7** and CB7 were prepared in DMSO and water respectively, which were diluted in media to prepare different concentrations. After 24 h, cells were incubated with different concentrations of free **aCy7**, free CB7, or **CB7@aCy7** (0–300 µM) for 72 h at 37 °C. Subsequently, the media above the cells were removed, and a fresh medium containing 3-(4,5-dimethylthiazol-2-yl)-2,5-diphenyltetrazolium bromide (MTT) was added to the cells. After incubation for 4 h, SDS-HCl was added to the wells to dissolve MTT crystals, and the cells were incubated overnight at 37 °C. The absorbance at 570 nm was measured, and a plot of cell metabolic activity was constructed.

## 4. Conclusions

This study described two new azobenzene–cyanine conjugates, **aCy7** and **aCy7s**, with NIR cyanine absorption and fluorescence emission maxima bands. Both conjugates exist as dispersed monomeric molecules in methanol, but they form non-emissive aggregates in water. The addition of CB7 to the dye solutions in water induces dye deaggregation and a substantial increase in cyanine NIR fluorescence emission intensity due to the formation of CB7 inclusion complexes with *K_1_*∼10^5^ M^−1^. CB7 encapsulates the protonated azonium tautomer of the 4-(*N*,*N*-dimethylamino)azobenzene component of the azobenzene–cyanine conjugate (Scheme 3) and produces a distinctive new absorption band at 530 nm. Methyl β-CD and albumin protein also capture **aCy7** or **aCy7s** and promote dye deaggregation, but they do not stabilize the 4-(*N*,*N*-dimethylamino)azobenzene component as its azonium tautomer.

The **CB7@aCy7** complex is quite hydrophilic, which suggests that CB7 can be used as a supramolecular additive to solubilize this new family of NIR azobenzene–cyanine conjugates for future biomedical applications. In particular, the azobenzene–cyanine conjugates have promise as “bioresponsive” fluorescent probes that “turn on” NIR cyanine fluorescence after the azobenzene component is cleaved by enzymes to produce a highly fluorescent heptamethine cyanine dye with a 4′-carboxyl group [29]. This future direction might require the modification of the conjugate structure to enhance the kinetic and thermodynamic stability of the CB7 inclusion complex in high-salt buffer solutions.

From a supramolecular perspective, the CB7 complexation of azobenzene compounds is emerging as a structurally rich topic with the complexes adopting a range of subtly different co-conformations [17,18,19,20,21]. Many azobenzene compounds are potential drug candidates or theranostic agents [22,23,24,25,26,61] but they are often not water-soluble [27]. It should be possible to formulate many of them as CB7 inclusion complexes with enhanced water solubility for more convenient dosage and improved pharmaceutical profile.

## Figures and Tables

**Table 1:** Photophysical properties of **aCy7** and **aCy7s** in methanol at room temperature.

Compound	Solvent	λ^abs^_max_ (nm)	λ^em^_max_ (nm)	Stokes Shift (nm)	*Φ_f_* (%) ^a^	ε (M^−1^ cm^−1^)
**aCy7**	MeOH	780	818	38	0.30	130,410
**aCy7s**	MeOH	786	820	34	0.36	173,700

^a^ Fluorescence quantum yield measurements used dye **1k** from [31] as a relative standard (*Φ_ref_* = 13.2% in methanol).

## Data Availability

The data presented in this study are available in Supplementary Materials.

## Supplementary Materials

The following supporting information can be downloaded at https://www.mdpi.com/article/10.3390/molecules27175440/s1, Figure S1. Absorption and emission spectra of 4.5 µM C1 with increasing concentrations of CB7 (0.0 eq–95.0 eq) in H2O, pH 5.70 at room temperature. (λex = 710 nm, Slit width = 5 nm). Figure S2. Representative titration of 4.5 μM C1 with increasing equivalents of CB7 in H2O, with the decrease in C1 absorption at 740 nm fitted to a 1:1 binding model1,2 (equation 2.7, see below).The same K1 value was obtained by fitting the data using Bindfit software. Figure S3. Absorption spectra in methanol/water mixtures containing (a) aCy7 (5 µM) or (b) aCy7s (4.5 µM). In 100% H2O, the absorption maxima for both the dyes indicates H-aggreagtes. Figure S4. (a, b) Absorption spectra of 5 µM aCy7 and 4.5 µM aCy7s in DCM, MeOH, DMSO or H2O. (c) Absorption spectra of 3.2 µM aCy7s in mixtures of DCM and MeOH. Figure S5. Dynamic light scattering (DLS) of aCy7 and aCy7s in water indicates aCy7 forms larger aggregates than aCy7s as indicated by the Zavg, the intensity weighted mean hydrodynamic size. Polydispersity Index (PDI) is a measure of the heterogeneity of a sample based on size. Figure S6. HRMS (ESI-TOF) spectra of aCy7 + CB7 (10 molar equivalents of CB7 added to 20 μM aCy7 in H2O) produced peaks for the 1:1 complex. Peaks corresponding to a 2:1 complex were not detected. Figure S7. HRMS (ESI-TOF) spectra of aCy7s + CB7 (30 molar equivalents of CB7 added to 10 μM aCy7s in H2O) produced peaks for 1:1 complex. Peaks corresponding to a 2:1 complex were not detected. Figure S8. Absorption spectra of 10 µM aCy7s with increasing amounts of CB7 (0.0 eq–40.0 eq) in H2O at room temperature. Upon the addition of CB7, the absorptions at 434 nm and 654 nm decrease and the absorptions at 535 nm and 780 nm increase, but there is no isosbestic point. (Change in pH: ∼pH 5.72 to pH 4.80) Figure S9. Absorption spectra of CB7@aCy7 complex in H2O with increasing dye concentrations at room temperature. Each sample contained 30 molar equivalents of CB7 to ensure complete formation of complex. Insert: linear Beer Lambert fit indicates negligible self-aggregation of the complex. Figure S10. Pictures of 10 μM free dye (aCy7 or aCy7s) and their CB7 complexes (30 molar equivalents of host) in H2O at room temperature. Figure S11. Absorption (left) and Emission (right) spectra of aCy7 before (blue, 8.5 µM) and after the addition of alpha-CD (red, 30 molar equivalents of alpha-CD) in H2O at room temperature. λex = 720 nm, Slit width = 5 nm. Figure S12. Absorption (left) and Emission (right) spectra of aCy7 before (blue, 8.5 µM) and after the addition of beta-CD (red, 30 molar equivalents of beta-CD) in H2O at room temperature. λex = 720 nm, Slit width = 5 nm. Figure S13. Absorption (left) and Emission (right) spectra of aCy7 before (blue, 8.5 µM) and after the addition of gamma-CD (red, 30 molar equivalents of gamma-CD) in H2O at room temperature. λex = 720 nm, Slit width = 5 nm. Figure S14. Absorption (left) and Emission (right) spectra of aCy7 before (blue, 8.5 µM) and after the addition of 2-hydroxypropyl beta-CD (red, 30 molar equivalents of 2-hydroxypropyl beta-CD) in H2O at room temperature. λex = 720 nm, Slit width = 5 nm. Figure S15. Absorption (left) and Emission (right) spectra of aCy7 before (blue, 8.5 µM) and after the addition of methyl beta-CD (red, 30 molar equivalents of methyl beta-CD) in H2O at room temperature. λex = 720 nm, Slit width = 5 nm. Figure S16. Absorption (left) and Emission (right) spectra of aCy7s before (blue, 10 µM) and after the addition of alpha-CD (red, 30 molar equivalents of alpha-CD) in H2O at room temperature. λex = 720 nm, Slit width = 5 nm. Figure S17. Absorption (left) and Emission (right) spectra of aCy7s before (blue, 10 µM) and after the addition of beta-CD (red, 30 molar equivalents of beta-CD) in H2O at room temperature. λex = 720 nm, Slit width = 5 nm. Figure S18. Absorption (left) and Emission (right) spectra of aCy7s before (blue, 10 µM) and after the addition of gamma-CD (red, 30 molar equivalents of gamma-CD) in H2O at room temperature. λex = 720 nm, Slit width = 5 nm. Figure S19. Absorption (left) and Emission (right) spectra of aCy7s before (blue, 10 µM) and after the addition of 2-hydroxypropyl beta-CD (red, 30 molar equivalents of 2-hydroxypropyl beta-CD) in H2O at room temperature. λex = 720 nm, Slit width = 5 nm. Figure S20. Absorption (left) and Emission (right) spectra of aCy7s before (blue, 10 µM) and after the addition of methyl beta-CD (red, 30 molar equivalents of methyl beta-CD) in H2O at room temperature. λex = 720 nm, Slit width = 5 nm. Figure S21. Absorption (left) and Emission (right) spectra of aCy7 before (blue, 10 µM) and after the addition of BSA (red, 30 molar equivalents of BSA) in H2O at room temperature. λex = 720 nm, Slit width = 5 nm. Figure S22. Absorption (left) and Emission (right) spectra of aCy7s before (blue, 10 µM) and after the addition of BSA (red, 30 molar equivalents of BSA) in H2O at room temperature. λex = 720 nm, Slit width = 5 nm. Figure S23. Cell metabolic activity (MTT assay) for MDA-MB-231 (adenocarcinoma) cells treated with varying concentrations (0–300 µM) of (a) free aCy7, (b) mixture CB7 + aCy7, or (c) free CB7. ^1^H- and ^13^C-NMR of compound **1**, **2**, **acy7** and **aCy7s**. DFT calculated molecular model of CB7@aCy7. References [27,28,62,63,64] are cited in the supplementary materials.

## Sample Availability

Not available.
