# Supplementary material for: Cucurbit[7]uril Complexation of Near-Infrared Fluorescent Azobenzene-Cyanine Conjugates
Source: Molecules. 2022 Aug 25;27(17):5440. doi: 10.3390/molecules27175440 (PMC9457616; doi:10.3390/molecules27175440)
Supplement: Supplementary file 1 [file molecules-27-05440-s001.zip › molecules-1880223-supplementary.pdf]

Supplementary Materials for:

Cucurbit[7]uril Complexation of Near-Infrared Fluorescent Azobenzene-Cyanine Conjugates

Sai Shradha Reddy Kommidi and Bradley D. Smith*

Department of Chemistry and Biochemistry, 251 Nieuwland Science Hall,

University of Notre Dame, Notre Dame, Indiana 46556, USA

*E-mail: smith.115@nd.edu

Contents

1. Weak binding of control dye C1 by CB7……………………………………………………….S2

2. Solvatochromism studies………………………………………………………………………S3

3. Dynamic light scattering……………………………………………………………………..S4

4. Binding studies with CB7

i) HRMS (ESI-TOF) spectra of CB7+aCy7 and CB7+aCy7s…………………………………S5

ii) Titration of aCy7s with CB7…………………………………………………………………S6

iii) Table S1: Spectral properties of CB7/dye complexes…………………………………...S6

iv) Photographs of CB7/dye complexes………………………………………………………..S7

5. Method for calculation of association constants for CB7@aCy7....................................S8

6. Binding of aCy7 or aCy7s by cyclodextrins (CDs)

i) Binding studies with aCy7……………………………………………………………………S10

ii) Binding studies with aCy7s………………………………………………………………....S12

7. Binding of aCy7 or aCy7s by bovine serum albumin (BSA)………………………………S14

8. Cell metabolic activity assay…………………………………………………………………S15

9. Synthesis and characterization……………………………………………………………...S16

10. DFT calculated molecular model of CB7@aCy7...S26

1. Weak binding of control dye C1 by CB7

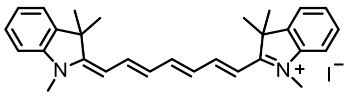

C1 (control heptamethine cyanine dye that lacks an appended 4-(N,N-dimethylamino)azobenzene component)

Titration experiment: A solution of 4.5 μM **C1** dye in H_2O was placed in a 1 mL quartz cuvette and titrated with aliquots from a CB7 stock solution (1.0 mM CB7 and 4.5 μM dye in H_2O). After each addition, the solution was allowed to equilibrate for 5 minutes, then an absorption and emission spectra was acquired. The decrease in the absorbance intensity at 740 nm (Figure S1) generated an isotherm that was fitted to a 1:1 binding model using Origin 8.6 software (Figure S2).

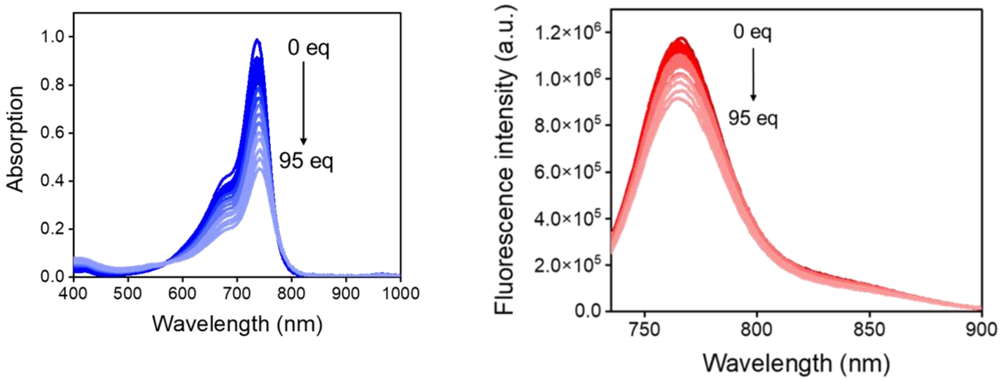

Figure S1. Absorption and emission spectra of 4.5 μM **C1** with increasing concentrations of CB7 (0.0 eq – 95.0 eq) in H_2O, pH 5.70 at room temperature. (λ_{ex} = 710 nm, Slit width = 5 nm)

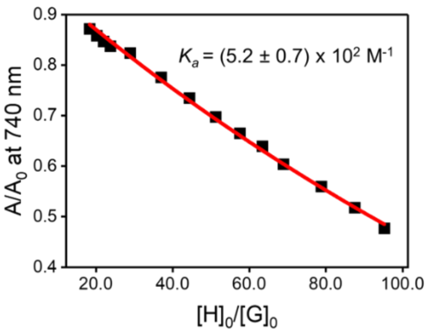

Independent variable	Regular residual of A/A_0
18.34862	-0.009033
20.20202	-0.010952
22.02202	-0.011423
23.80952	-0.010104
28.98551	0.0071815
37.03704	0.0052466
44.44444	0.0052995
51.28205	0.0042611
57.61317	0.0047672
63.49206	0.0083573
68.96552	-1.00E-03
78.85305	0.0019558
87.54209	-9.95E-04
95.2381	-0.008407

Figure S2. Representative titration of 4.5 μM **C1** with increasing equivalents of CB7 in H_2O, with the decrease in **C1** absorption at 740 nm fitted to a 1:1 binding model[1,2] (equation 2.7, see below). The same K_1 value was obtained by fitting the data using Bindfit software.[3] The Bindfit data and output file can be accessed at: http://app.supramolecular.org/bindfit/view/73e50699-0ce9-41af-a520-4dc0863fffbf

2. Solvatochromism studies

(a)

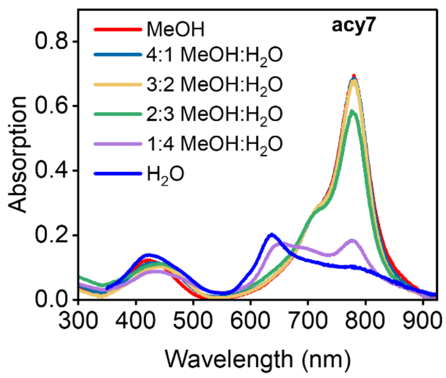

(b)

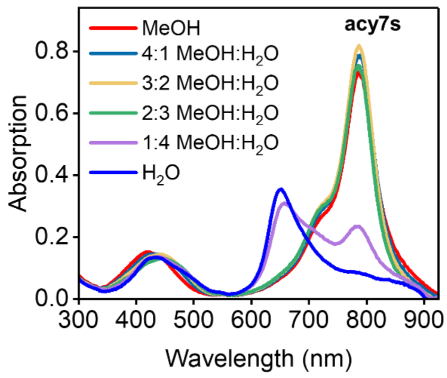

Figure S3. Absorption spectra in methanol/water mixtures containing (a) **aCy7** (5 µM) or (b) **aCy7s** (4.5 µM). In 100% H_2O, the absorption maxima for both the dyes indicates H-aggreagtes.

(a)

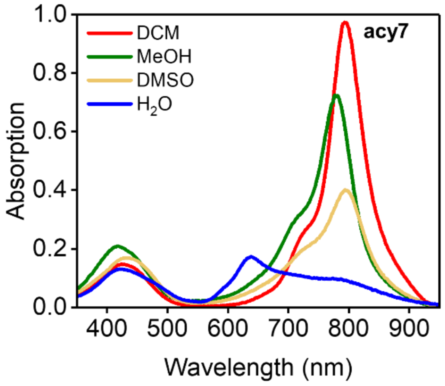

(b)

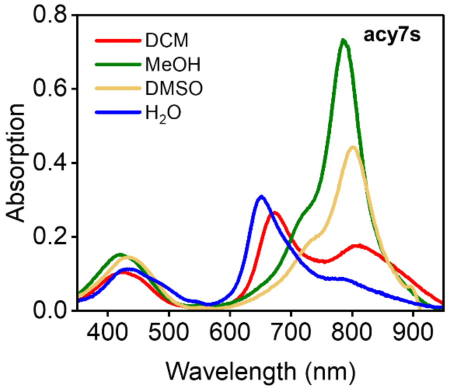

(c)

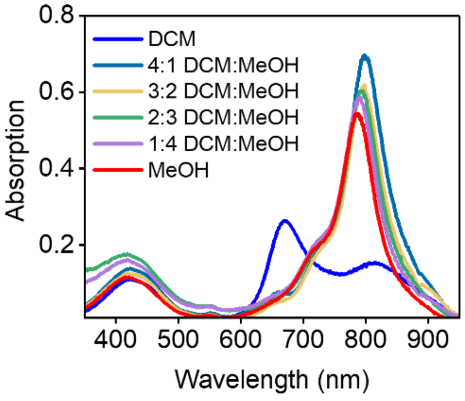

Figure S4. (a, b) Absorption spectra of 5 µM **aCy7** and 4.5 µM **aCy7s** in DCM, MeOH, DMSO or H_2O. (c) Absorption spectra of 3.2 µM **aCy7s** in mixtures of DCM and MeOH.

3. Dynamic light scattering

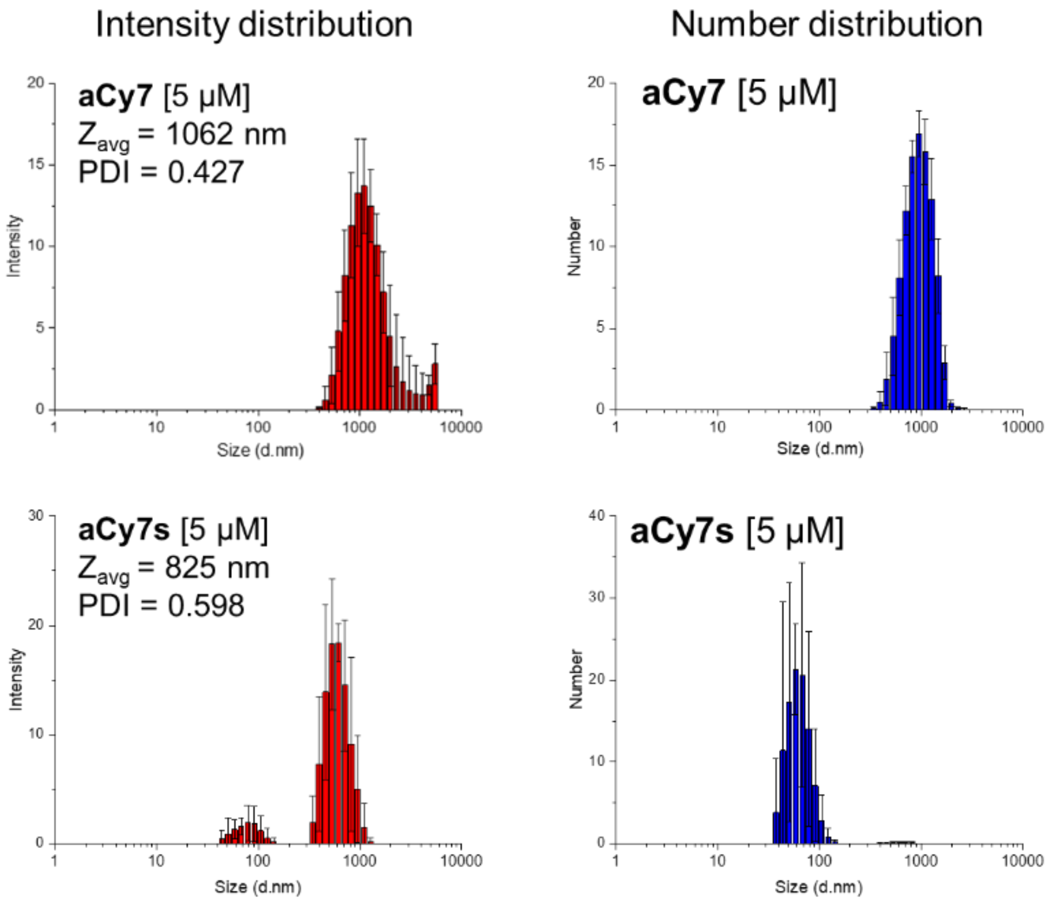

Figure S5. Dynamic light scattering (DLS) of **aCy7** and **aCy7s** in water indicates **aCy7** forms larger aggregates than **aCy7s** as indicated by the Z_{avg}, the intensity weighted mean hydrodynamic size. Polydispersity Index (PDI) is a measure of the heterogeneity of a sample based on size.

4. Binding studies with CB7

i. HRMS (ESI-TOF) spectra of CB7+aCy7 and CB7+aCy7s

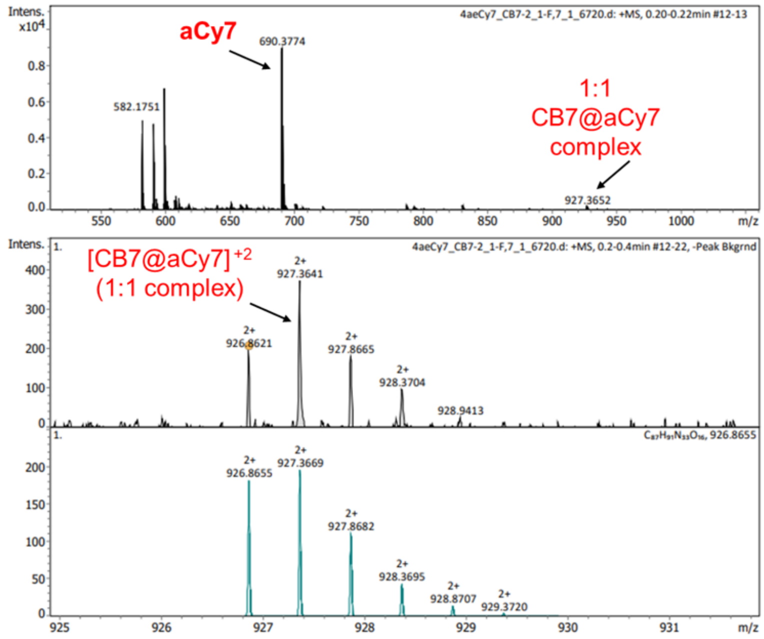

Figure S6. HRMS (ESI-TOF) spectra of **aCy7** + CB7 (10 molar equivalents of CB7 added to 20 μM **aCy7** in H_2O) produced peaks for the 1:1 complex. Peaks corresponding to a 2:1 complex were not detected.

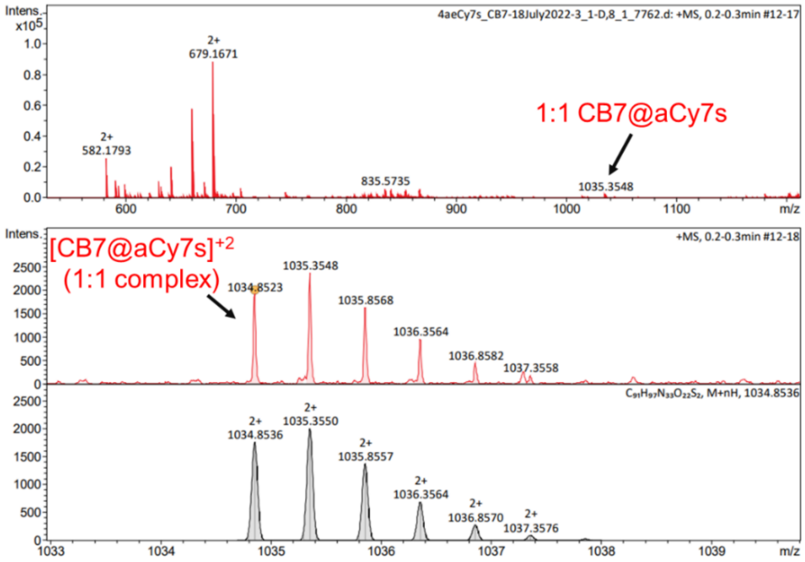

Figure S7. HRMS (ESI-TOF) spectra of **aCy7s** + CB7 (30 molar equivalents of CB7 added to 10 μM **aCy7s** in H_2O) produced peaks for 1:1 complex. Peaks corresponding to a 2:1 complex were not detected.

ii. Titration of aCy7s with CB7

A solution of 10 μM **aCy7s** in H_2O was placed in a 1 mL quartz cuvette and titrated with aliquots from a CB7 stock solution (1.0 mM CB7 and 10 μM dye in H_2O). The solution was allowed to equilibrate for 5 minutes. Following each addition of the host, an absorption spectrum was acquired.

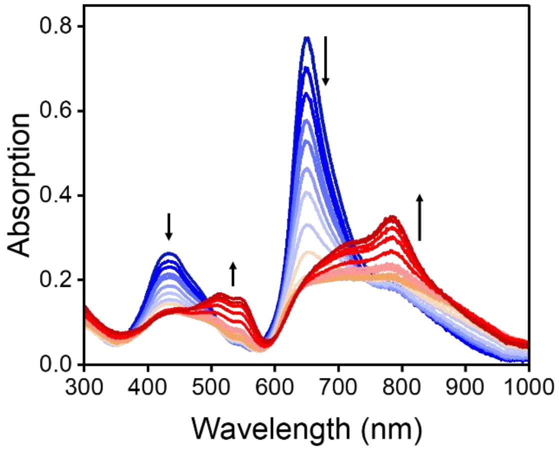

Figure S8. Absorption spectra of 10 μM **aCy7s** with increasing amounts of CB7 (0.0 eq - 40.0 eq) in H_2O at room temperature. Upon the addition of CB7, the absorptions at 434 nm and 654 nm decrease and the absorptions at 535 nm and 780 nm increase, but there is no isosbestic point. (Change in pH: ~pH 5.72 to pH 4.80)

iii. Table S1: Spectral properties of CB7 complexes of **aCy7** and **aCy7s** in H_2O at room temperature

Compound	Solvent	λ^{abs}_{max} (nm)	λ^{em}_{max} (nm)	Stokes shift (nm)	ε (M^{-1} cm^{-1})
**CB7@aCy7**[a]	H_2O	782	813	31	92,160
**CB7@aCy7s**[a, b]	H_2O	784	818	33	-

[a] Solution contained dye + 30 molar equivalents of CB7

[b] Molar absorptivity for **CB7@aCy7s** complex could not be calculated, as H-aggregate band at 654 nm was still present.

Molar absorptivity of CB7@aCy7

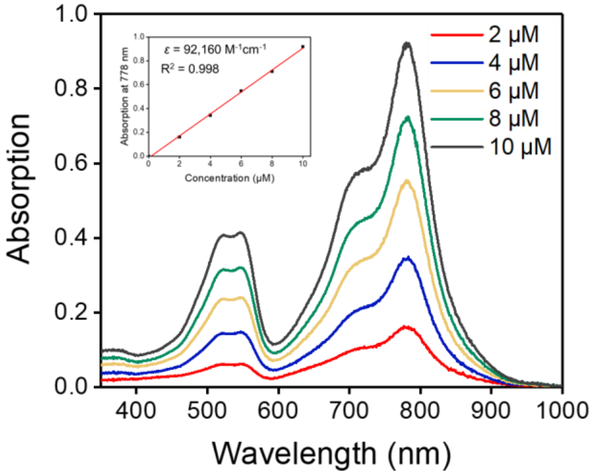

Figure S9. Absorption spectra of **CB7@aCy7** complex in H_2O with increasing dye concentrations at room temperature. Each sample contained 30 molar equivalents of CB7 to ensure complete formation of complex. Insert: linear Beer Lambert fit indicates negligible self-aggregation of the complex.

iv. Photographs of CB7/dye complexes

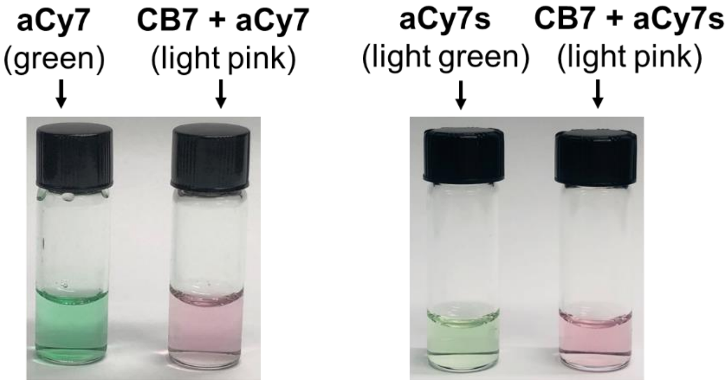

Figure S10. Pictures of 10 μM free dye (**aCy7** or **aCy7s**) and their CB7 complexes (30 molar equivalents of host) in H_2O at room temperature.

5. Calculation of association constants

Algorithm for Determining K_a [1,2]

For a 1:1 host-guest system:

$$H + G \underset{}{\overset{K_a}{\rightleftharpoons}} HG$$

The desired association constant is expressed in equation (1.1):

K_a = [HG]/[H][G]

Assigning the total concentration of host and guest as $[H]_0$ and $[G]_0$, respectively, gives mass balance equations (1.2) and (1.3)

$[H]_0$ = [H] + [HG] (1.2)

$[G]_0$ = [G] + [HG] (1.3)

Equation (1.2) is rearranged to define [H] and equation (1.3) is rearranged to define [G]. The newly defined [H] and [G] are used to replace [H] and [G] in equation (1.1) and the resulting equation is rearranged to yield equation (1.4):

$[HG]^2 - ([H]_0 + [G]_0 + 1/K_a)[HG] + [H]_0[G]_0 = 0$ (1.4)

The real root of equation (1.4) is expressed in equation (1.5), which defines [HG] based on K_a and experimentally determined values ($[H]_0$ and $[G]_0$):

$[HG] = 0.5*(([H]_0 + [G]_0 + 1/K_a) - \text{sqrt}\{([H]_0 + [G]_0 + 1/K_a)^2 - 4[H]_0[G]_0\})$ (1.5)

In our analysis, we treated either G_0 or H_0 as slightly adjustable to reach the best fitting, since real molar concentration is inherently dependent on a variety of experimental conditions (i.e., polydispersity of dye, balance error, photobleaching of cyanine dye). Varying this parameter, and related values in later analyses, greatly improves the accuracy of the fitting result. We define the host-guest ratio as:

$[H]_0/[G]_0$ = r (1.6).

We further introduce the concentration correction coefficient m into equation (1.6):

$[H]_0/(m[G]_0)$ = r (1.7).

According to Beer's law,

A = ε_Hb[H]+ε_Gb[G]+ε_{HG}b[HG] (2.1), where ε_H and ε_G are the molar absorptivity of host and guest respectively, and b is the path length (10 mm).

If at the selected wavelength, ε_H=0, then equation (2.1) can be simplified as equation (2.2):

A = ε_Gb[G]+ε_{HG}b[HG] (2.2)

According to equation (2.3), [G] can be defined by $[G]_0$ and [HG], thus (2.2) gives equation (2.3):

$A = \varepsilon_G b[G]_0 + (\varepsilon_{HG} - \varepsilon_G)b[HG]$ (2.3).

At the beginning of the titration,

$A_0 = \varepsilon_G b[G]_0$ (2.4), then

$\Delta A = A - A_0 = (\varepsilon_{HG} - \varepsilon_G)b[HG] = \varepsilon b[HG]$ (2.5), where $\varepsilon = \varepsilon_{HG} - \varepsilon_G$

From Equation (1.5) and (2.5), we get

$\Delta A = f([H]_0, [G]_0, \varepsilon, K_a)$ (2.6)

Here we established the relationship between absorption data ΔA, the concentrations $[H]_0$ and $[G]_0$, the absorption coefficients ε_{HG} and ε_G, and the association constant K_a. From equations (2.6) and (1.7), we get

$\Delta A = f([G]_0, r, m, \varepsilon, K_a)$ (2.7),

which was used for the nonlinear fitting in Origin 8.6 to obtain the association constant K_a.

To get an approximate association constant for the **CB7@aCy7** complex, both the decrease in absorption at 434 nm (corresponding to the non-protonated azonium of azobenzene) and at 645 nm (corresponding to the cyanine H-aggregate) were then plotted to produce isotherms that were fitted to equation 2.7 using Origin 8.6.

Below are the independent variables and residual A/A_0 values for the binding curves in Figure 4.

Decrease in absorbance at 434 nm

Independent Variable	Regular Residual of A/A_0
0	0
0.27701	-0.02143
0.55249	-0.00577
0.82645	0.00905
1.0989	0.0091
1.36986	0.00771
1.63934	3.44E-04
1.87556	0.00327
2.17391	0.00409
2.43902	-0.00537
2.66056	-0.01071
2.92398	-0.00976
3.26642	-0.0113
3.73531	0.00334
4.25532	0.00559
4.7619	0.0043
5.26316	0.00545

Decrease in absorbance at 635 nm

Independent Variable	Regular Residual of A/A_0
0	0
0.27701	-0.0133
0.55249	-0.00971
0.82645	0.00889
1.0989	3.59E-04
1.36986	0.01094
1.63934	0.01352
1.87556	0.00134
2.17391	-0.00582
2.43902	-0.01335
2.66056	-0.00533
2.92398	-0.00486
3.26642	-0.00151
3.73531	0.00146
4.25532	-0.00533
4.7619	0.00775
5.26316	0.00598

The same K_1 values were obtained by fitting the data using Bindfit software.[3]
The Bindfit data and output files can be accessed at:
434 nm: http://app.supramolecular.org/bindfit/view/f643d15a-2b00-46df-8628-7230a158ed89
635 nm: http://app.supramolecular.org/bindfit/view/a742fd99-a005-476e-bae5-b4dc2ce66bfb

6. Binding of aCy7 and aCy7s with Cyclodextrins (CDs)

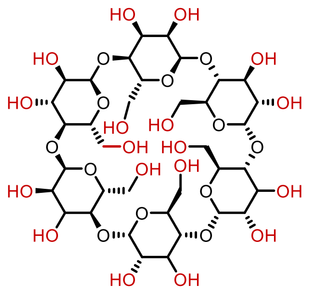

alpha-cyclodextrin

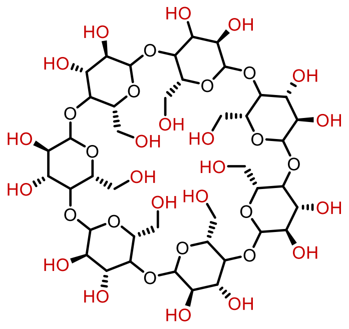

beta cyclodextrin

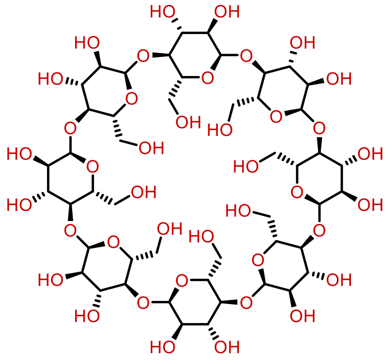

gamma cyclodextrin

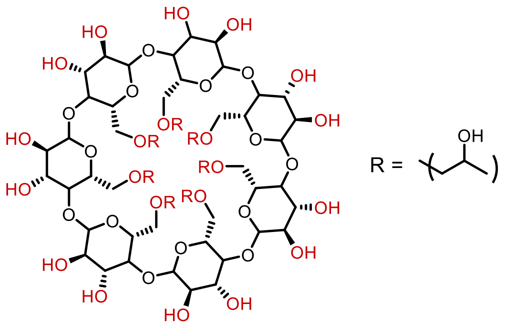

2-hydroxypropyl beta cyclodextrin

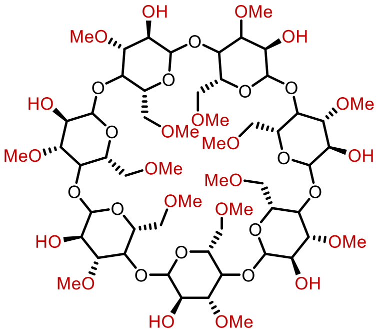

methyl beta cyclodextrin

Five different cyclodextrins (CDs): alpha-CD, beta-CD, gamma-CD, 2-hydroxypropyl beta CD or methyl beta CD, were added to separate solutions of **aCy7** or **aCy7s**.

i) Binding of aCy7 by cyclodextrins (CDs)

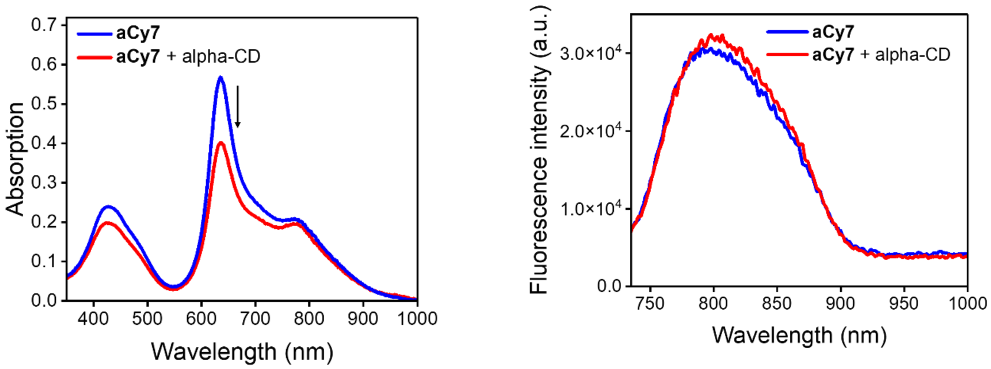

Figure S11. Absorption (left) and Emission (right) spectra of **aCy7** before (blue, 8.5 µM) and after the addition of alpha-CD (red, 30 molar equivalents of alpha-CD) in H_2O at room temperature. λ_{ex} = 720 nm, Slit width = 5 nm.

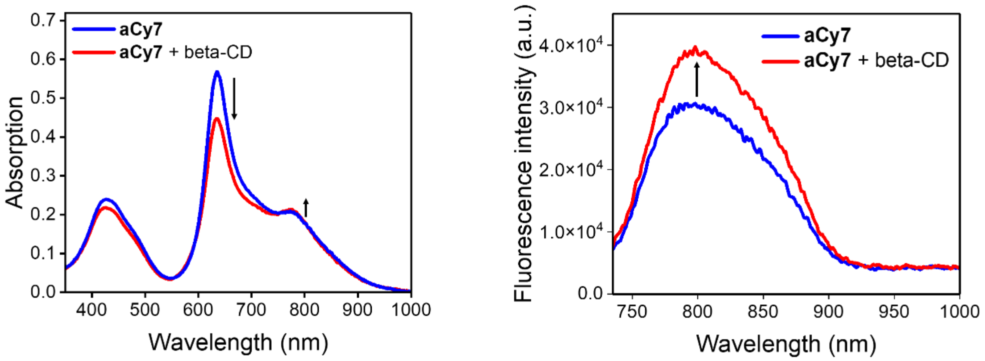

Figure S12. Absorption (left) and Emission (right) spectra of **aCy7** before (blue, 8.5 µM) and after the addition of beta-CD (red, 30 molar equivalents of beta-CD) in H_2O at room temperature. λ_{ex} = 720 nm, Slit width = 5 nm.

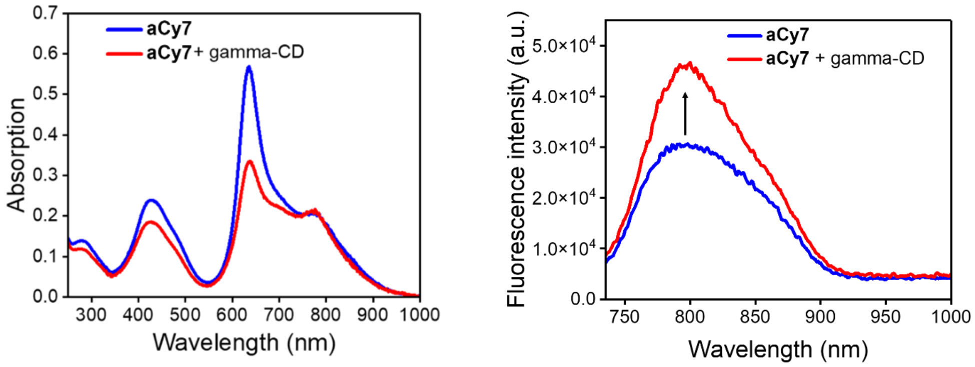

Figure S13. Absorption (left) and Emission (right) spectra of **aCy7** before (blue, 8.5 µM) and after the addition of gamma-CD (red, 30 molar equivalents of gamma-CD) in H_2O at room temperature. λ_{ex} = 720 nm, Slit width = 5 nm.

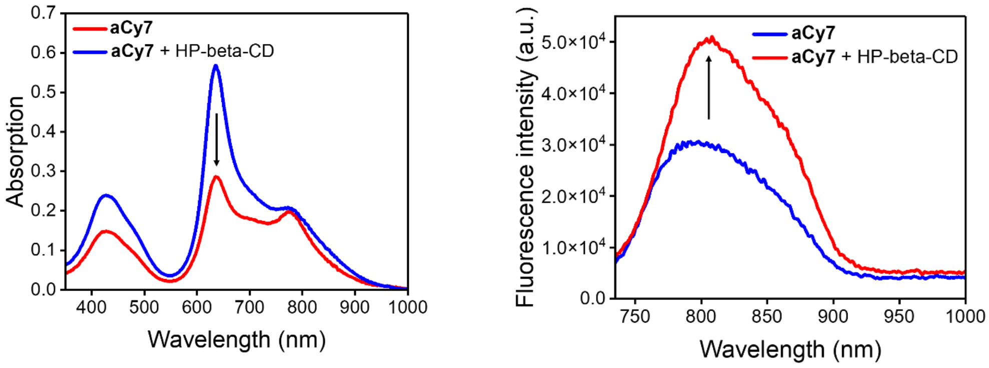

Figure S14. Absorption (left) and Emission (right) spectra of **aCy7** before (blue, 8.5 µM) and after the addition of 2-hydroxypropyl beta-CD (red, 30 molar equivalents of 2-hydroxypropyl beta-CD) in H_2O at room temperature. λ_{ex} = 720 nm, Slit width = 5 nm.

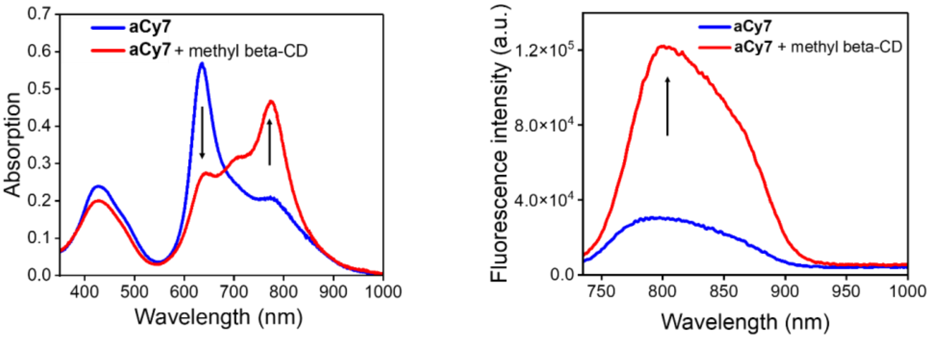

Figure S15. Absorption (left) and Emission (right) spectra of **aCy7** before (blue, 8.5 µM) and after the addition of methyl beta-CD (red, 30 molar equivalents of methyl beta-CD) in H_2O at room temperature. λ_{ex} = 720 nm, Slit width = 5 nm

ii) <u>Binding of aCy7s by cyclodextrins (CDs)</u>

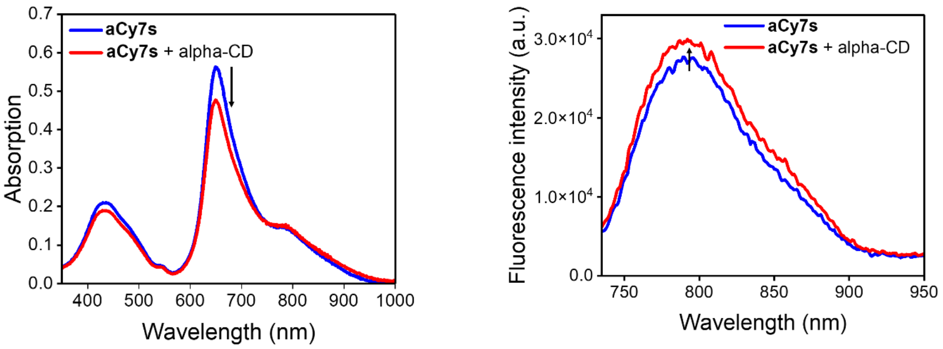

Figure S16. Absorption (left) and Emission (right) spectra of **aCy7s** before (blue, 10 µM) and after the addition of alpha-CD (red, 30 molar equivalents of alpha-CD) in H_2O at room temperature. λ_{ex} = 720 nm, Slit width = 5 nm

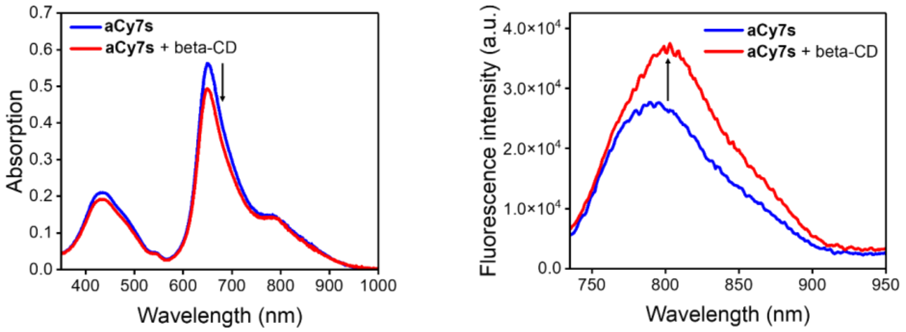

Figure S17. Absorption (left) and Emission (right) spectra of **aCy7s** before (blue, 10 µM) and after the addition of beta-CD (red, 30 molar equivalents of beta-CD) in H_2O at room temperature. λ_{ex} = 720 nm, Slit width = 5 nm.

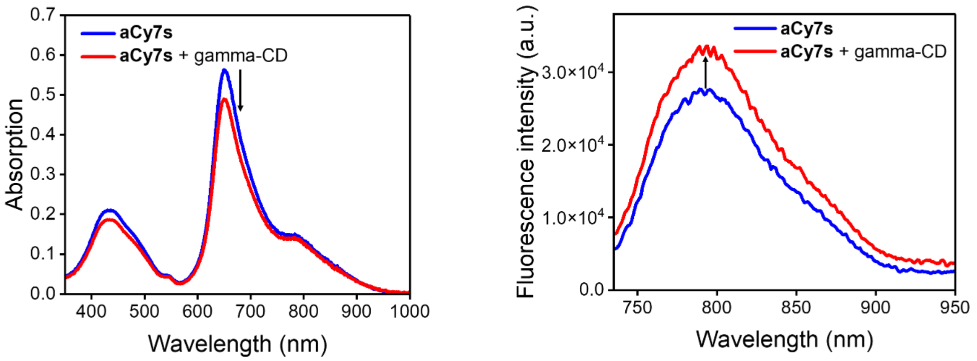

Figure S18. Absorption (left) and Emission (right) spectra of **aCy7s** before (blue, 10 μM) and after the addition of gamma-CD (red, 30 molar equivalents of gamma-CD) in H_2O at room temperature. λ_{ex} = 720 nm, Slit width = 5 nm.

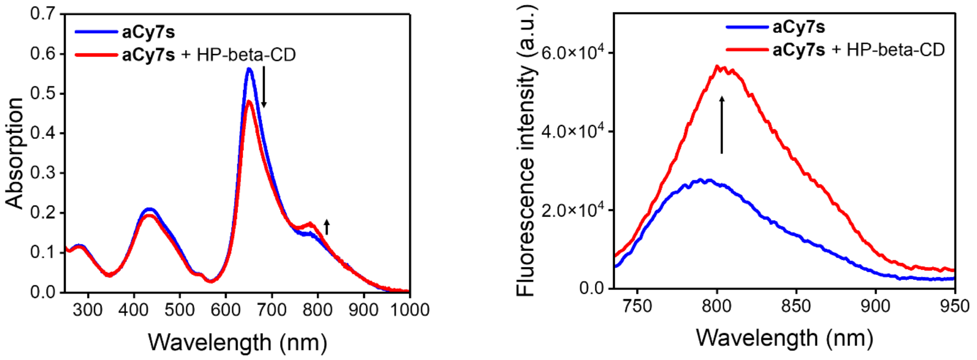

Figure S19. Absorption (left) and Emission (right) spectra of **aCy7s** before (blue, 10 μM) and after the addition of 2-hydroxypropyl beta-CD (red, 30 molar equivalents of 2-hydroxypropyl beta-CD) in H_2O at room temperature. λ_{ex} = 720 nm, Slit width = 5 nm.

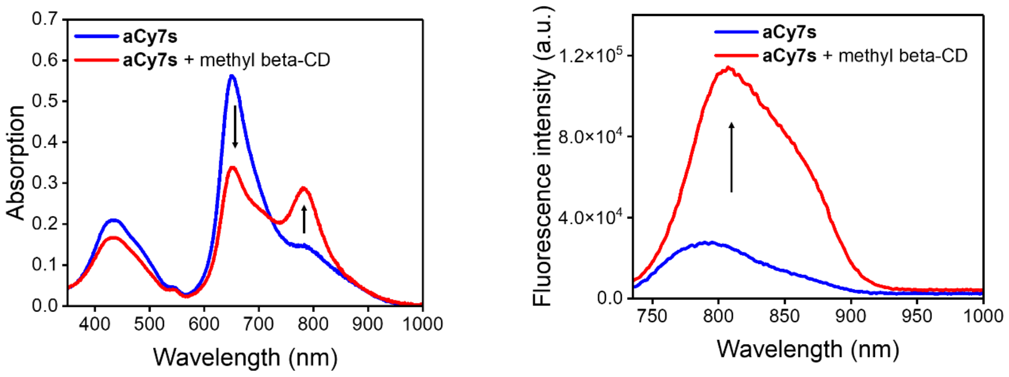

Figure S20. Absorption (left) and Emission (right) spectra of **aCy7s** before (blue, 10 μM) and after the addition of methyl beta-CD (red, 30 molar equivalents of methyl beta-CD) in H_2O at room temperature. λ_{ex} = 720 nm, Slit width = 5 nm.

7. Binding of aCy7 or aCy7s by bovine serum albumin (BSA)

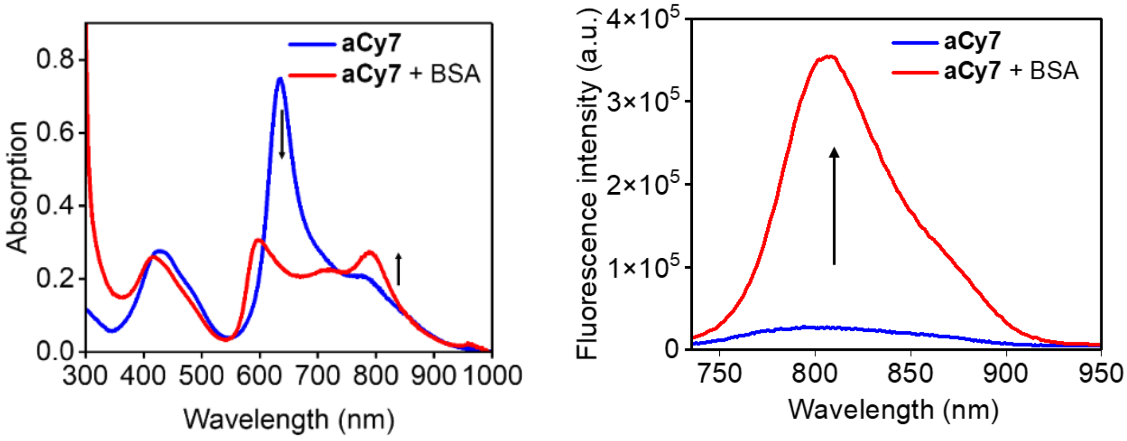

Figure S21. Absorption (left) and Emission (right) spectra of **aCy7** before (blue, 10 µM) and after the addition of BSA (red, 30 molar equivalents of BSA) in H_2O at room temperature. λ_{ex} = 720 nm, Slit width = 5 nm.

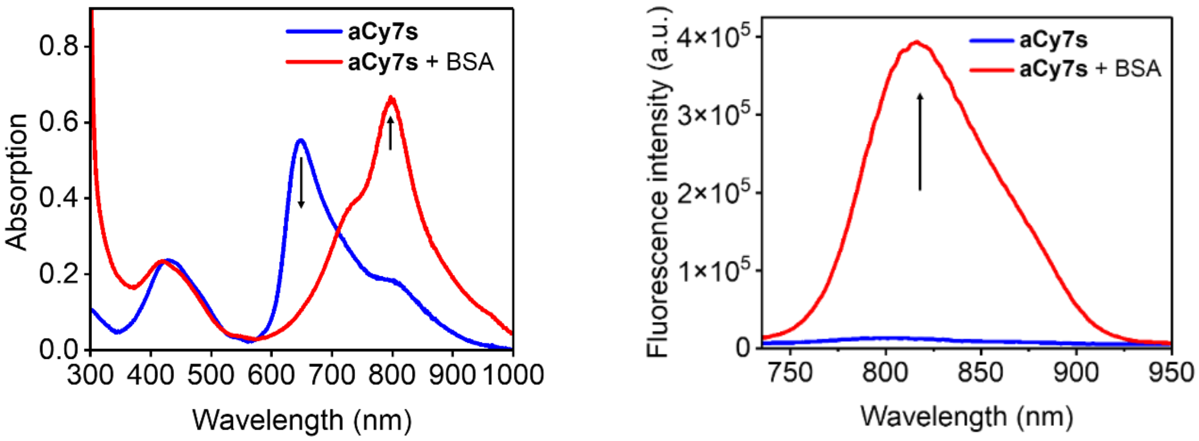

Figure S22. Absorption (left) and Emission (right) spectra of **aCy7s** before (blue, 10 µM) and after the addition of BSA (red, 30 molar equivalents of BSA) in H_2O at room temperature. λ_{ex} = 720 nm, Slit width = 5 nm.

8. Cell metabolic activity assay

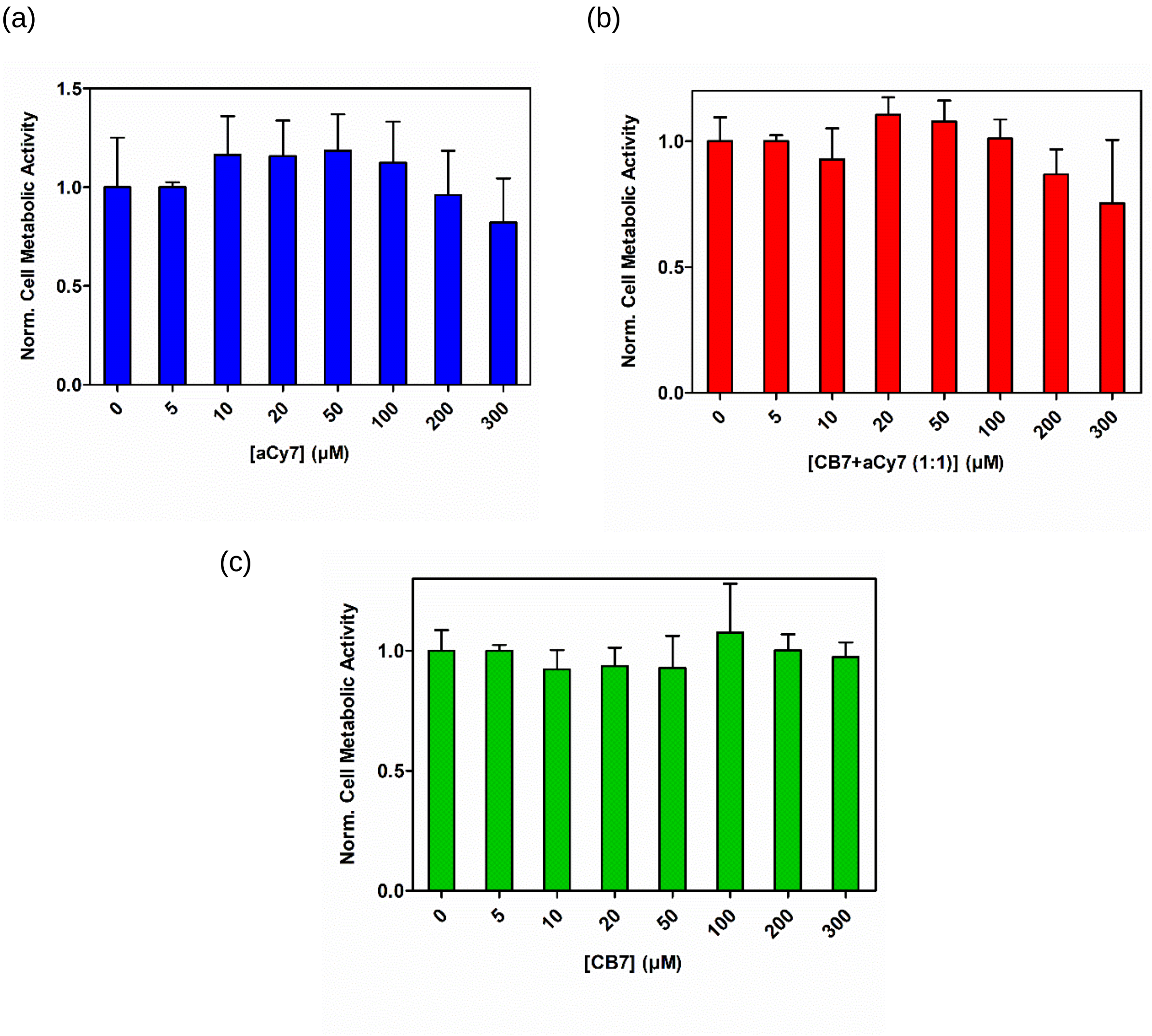

Figure S23. Cell metabolic activity (MTT assay) for MDA-MB-231 (adenocarcinoma) cells treated with varying concentrations (0 – 300 μM) of (a) free **aCy7**, (b) mixture CB7 + **aCy7**, or (c) free CB7.

9. Synthesis and characterization

Compound **1** was synthesized according to a reported procedure.[4]

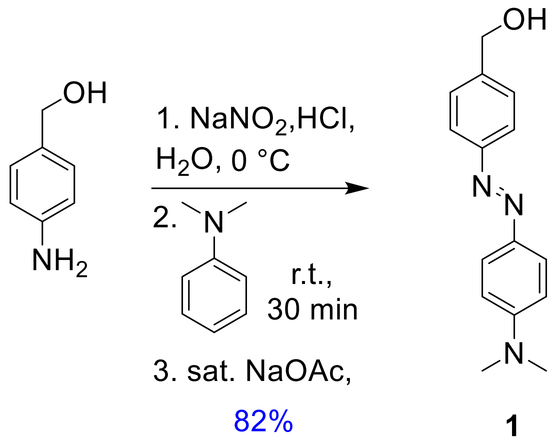

1

Compound **1**. 4-Aminobenzyl alcohol (3 g, 24.36 mmol, 1 eq) was suspended in 50 mL 4M HCl in a 500 mL round bottom flask. The solution was cooled to 0 ^{o}C in an ice-bath and sodium nitrite (2.1 g, 29.23 mmol, 1.2 eq) solution in distilled water (10 mL) was added dropwise to the reaction flask producing white vapors. The reaction mixture was stirred at 0 ^{o}C for 2 hours. Then, *N, N*-dimethylaniline (3.14 mL, 24.36 mmol, 1 eq) was added slowly and the reaction mixture was stirred for additional 30 min at room temperature. The reaction mixture was treated with 100 mL saturated sodium acetate solution in water to afford a brown precipitate. The precipitate was vacuum filtered and washed with 50 mL hot distilled water. The crude was purified through column chromatography (SiO_2, 0-25% EtOAc in Hexane) and dried *in vacuo* to afford compound **1** as an orange solid (5.1 g, 82%). ^{1}H NMR (400 MHz, $CDCl_3$, 25 °C) δ(ppm): 7.79 (q, *J* = 8.0 Hz, 4H), 7.39 (d, *J* = 8.0 Hz, 2H), 6.68 (d, *J* = 12.0 Hz, 2H), 4.68 (s, 2H), 3.02 (s, 6H).

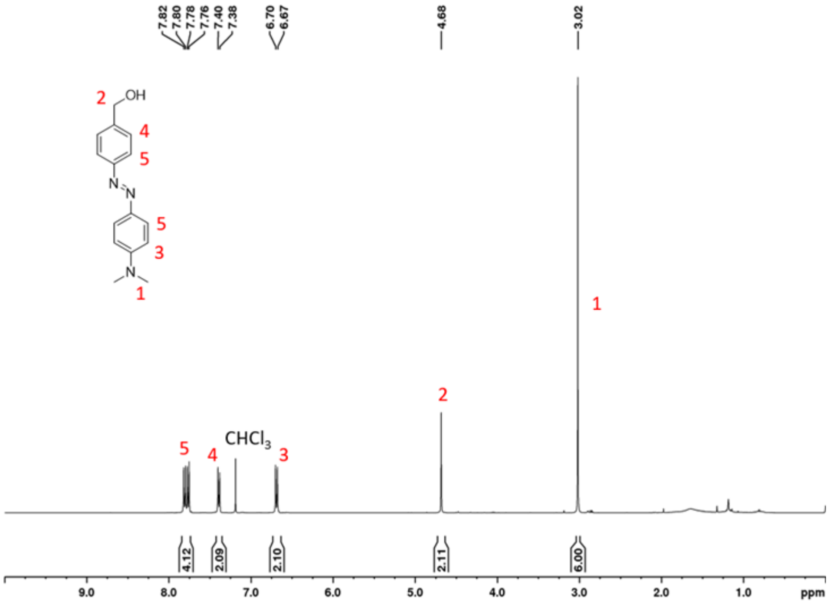

^{1}H NMR spectrum (400 MHz, $CDCl_3$, 25°C) of **1**

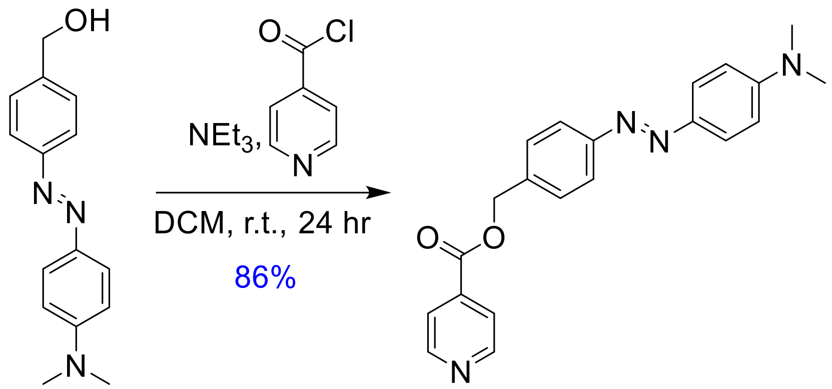

2

Compound **2**. A mixture of isonicotinic acid (2 g, 16.24 mmol, 1 eq) and thionyl chloride (7.10 mL, 97.47 mmol, 6 eq) was refluxed for 3 hours under an argon atmosphere. The thionyl chloride was evaporated under reduced pressure and dry DCM (25 mL) was added to the same flask. The alcohol **1** (4.57 g, 17.86 mmol, 1.1 eq) was added followed by dropwise addition of triethylamine (4.53 mL, 32.48 mmol, 2 eq) under an argon atmosphere. The reaction mixture was stirred overnight yielding an orange precipitate which was vacuum filtered and washed with DCM. The crude was purified through column chromatography (SiO_2, 25% EtOAc in Hexane) and dried *in vacuo* to afford compound **2** as a bright orange solid (5 g, 86%). ^{1}H NMR (400 MHz, methanol-*d*$_4$, 25 °C) δ(ppm): 8.74 (dd, *J* = 4.2 Hz, 2H), 7.98 (dd, *J* = 4.2, 2H), 7.82 (d, *J* = 8.5 Hz, 2H), 7.59 (d, *J* = 8.3 Hz, 2H), 6.82 (d, *J* = 9.8 Hz, 2H), 5.47 (s, 2H), 3.09 (s, 6H).

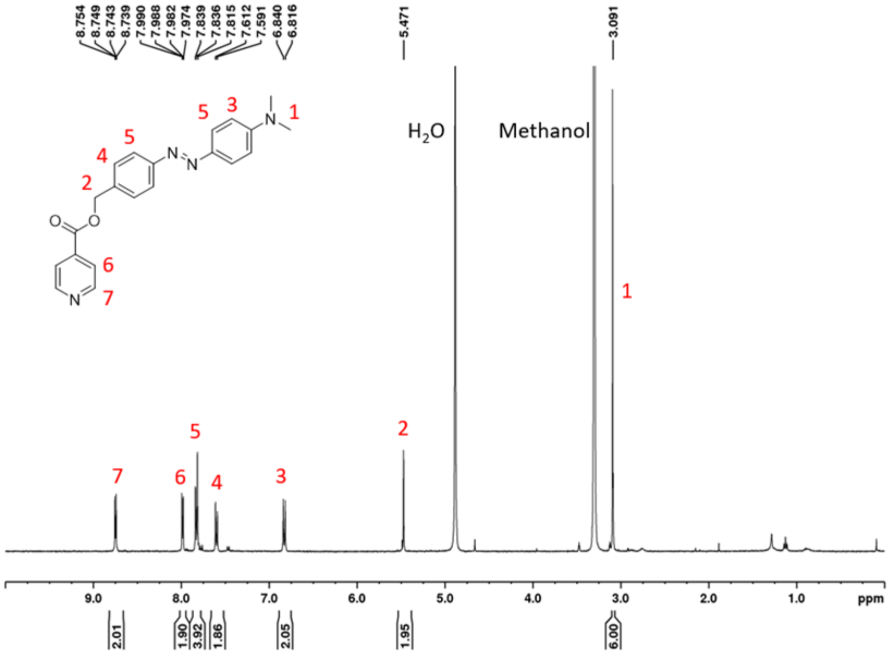

^{1}H NMR spectrum (400 MHz, methanol-*d*$_4$, 25°C) of **2**

Compounds **3**, **5** and **6** were synthesized according to a reported procedure.[5]

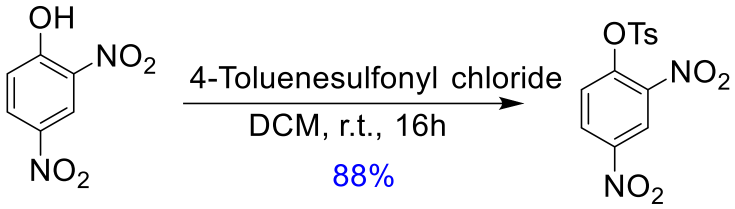

3

Compound **3**. To a mixture of 2,4-dinitrophenol (3.08 g, 16.73 mmol, 1 eq) and tosyl chloride (3.50 g, 18.40 mmol, 1.01 eq) in DCM (50 mL), triethylamine (5.83 mL, 41.82 mmol, 2.5 eq) was added and stirred overnight. The reaction mixture was washed with 50 mL of distilled water, and the organic layer extracted. The aqueous layer was washed three times with 50 mL portions of DCM, all the organic layers combined, washed with 50 mL $NaHCO_3$ and 50 mL brine. The organic layer was extracted, dried over $MgSO_4$, and evaporated under reduced pressure. A trituration was performed with hot (55°C) methanol to give the product, **3**, as a light-yellow powder (5 g, 88%). ^1H NMR (400 MHz, $CDCl_3$, 25 °C) δ(ppm): 8.70 (d, *J* = 2.8 Hz, 1H), 8.42 (dd, *J* = 9.1, 2.8 Hz, 1H), 7.73 (d, *J* = 8.4 Hz, 2H), 7.66 (d, *J* = 9.0 Hz, 1H), 7.33 (d, *J* = 8.1 Hz, 2H), 2.43 (s, 3H).

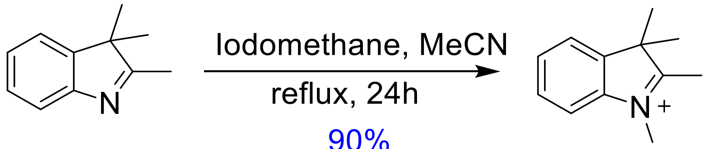

5

Compound **5**. 2,3,3-Trimethylindolenine (6.14 mL, 38.30 mmol, 1 eq) and iodomethane (4.8 mL, 76.6 mmol, 2 eq) were dissolved in acetonitrile (30 mL). The reaction mixture was refluxed overnight to yield a pink precipitate. The reaction was cooled to room temperature, the precipitate was filtered, washed with ethyl acetate (100 mL) and diethyl ether (50 mL), dried *in vacuo* to give **5** as a pink powdered solid (6 g, 90%). ^1H NMR (400 MHz, $CDCl_3$, 25 °C) δ(ppm): 7.49 – 7.58 (m, 4 H), 4.22 (s, 3H), 3.06 (s, 3H), 1.62 (s, 6H).

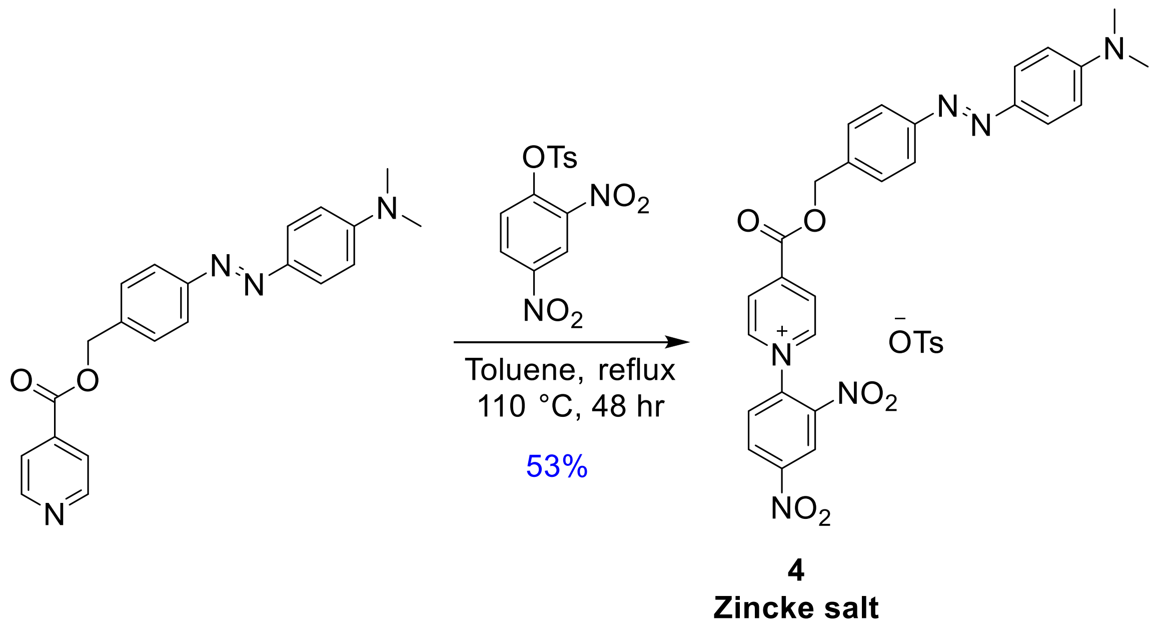

Compound **4**. A mixture of **2** (1g, 2.80 mmol, 1 eq) and **3** (2.1 g, 6.17 mmol, 2.2 eq) in toluene (20 mL) was refluxed for 48 hours yielding a reddish orange precipitate at the bottom of the flask. The reaction mixture was allowed to cool to room temperature and placed in a -20°C freezer for 15 minutes. The precipitate was vacuum filtered, washed with ethyl acetate (50 mL), and dried *in vacuo* to afford a reddish orange solid **4** (788 mg, 53%) which was used without purification in the next step.

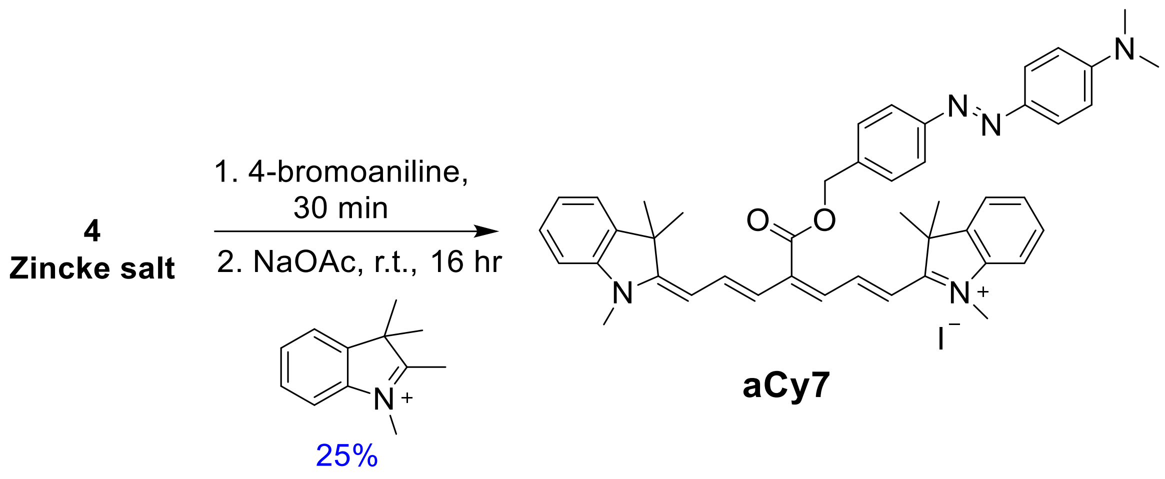

Compound **aCy7**. A mixture of Zincke salt **4** (300 mg, 0.57 mmol, 1 eq) and 4-bromoaniline (117.4 mg, 0.68 mmol, 1.2 eq) in methanol (15 mL) was stirred for 30 min at room temp, followed by the addition of indole salt **5** (247.8 mg, 1.42 mmol, 2.5 eq) and sodium acetate (233.3 mg, 2.84 mmol, 5 eq). The reaction mixture was stirred overnight at room temperature in the dark, turning a green color. Diethyl ether (100 mL) was added to the reaction mixture, and it was stored in a -20°C

freezer for 30 min. The green precipitate was collected by vacuum filtration and the filtered crude was purified by column chromatography (SiO_2, 0-5% MeOH in DCM) to afford **aCy7** as a green solid (98 mg, 25%). ^{1}H NMR (400 MHz, DMSO-d_6, 25 °C) δ(ppm): 7.85-7.80 (dd, *J* = 9.0 Hz, 4H), 7.80-7.77 (t, *J* = 13.6 Hz, 1H), 7.70-7.68 (d, *J* = 8.3 Hz, 2H), 7.50-7.42 (m, 6H), 7.23 (t, *J* = 7.6 Hz, 2H), 6.86 (d, *J* = 9.0 Hz, 2H), 6.68 (d, *J* = 13.9 Hz, 2H), 6.50 (d, *J* = 13.8 Hz, 2H), 5.55 (s, 2H), 3.62 (s, 6H), 3.08 (s, 6H), 1.47 (s, 12 H). ^{13}C NMR (126 MHz, DMSO-d_6, 25°C) δ (ppm): 172.5, 167.1, 153.1, 144.7, 143.03, 141.50, 137.0, 130.5, 129.0, 125.6, 125.4, 122.7, 112.0, 112.0, 107.0, 67.5, 49.1, 32.0, 27.4. HRMS (ESI-TOF) m/z: $[M + H]^+$ calcd for $C_{45}H_{48}N_5O_2^+$ 690.3808, found 690.3805.

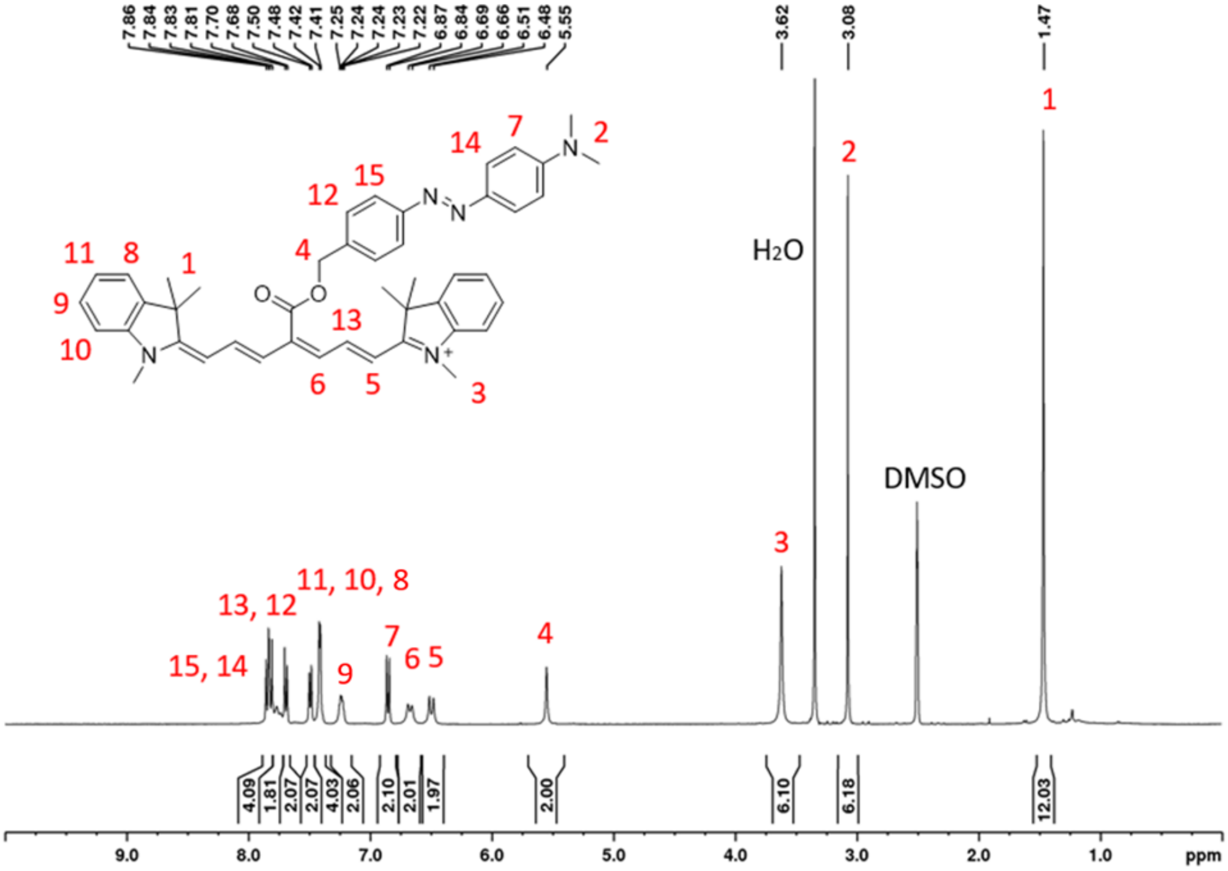

^{1}H NMR spectrum (400 MHz, DMSO-d6, 25°C) of **aCy7**

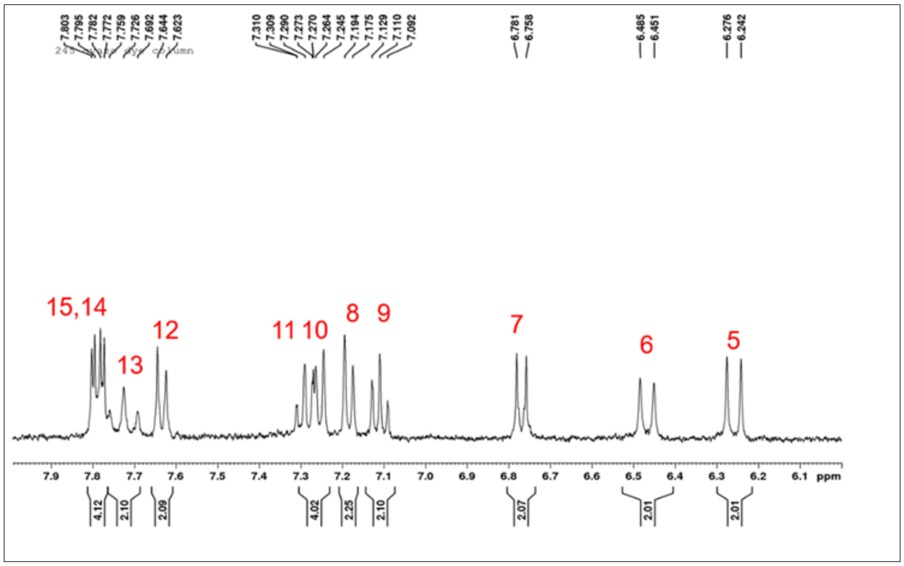

^{1}H NMR spectrum (400 MHz, methanol-d_4, 25°C) expanded aromatic region (6.5 - 8.5 ppm) of **aCy7**. The splitting pattern for **aCy7** in MeOD-d4 is more distinct than in DMSO-d_6.

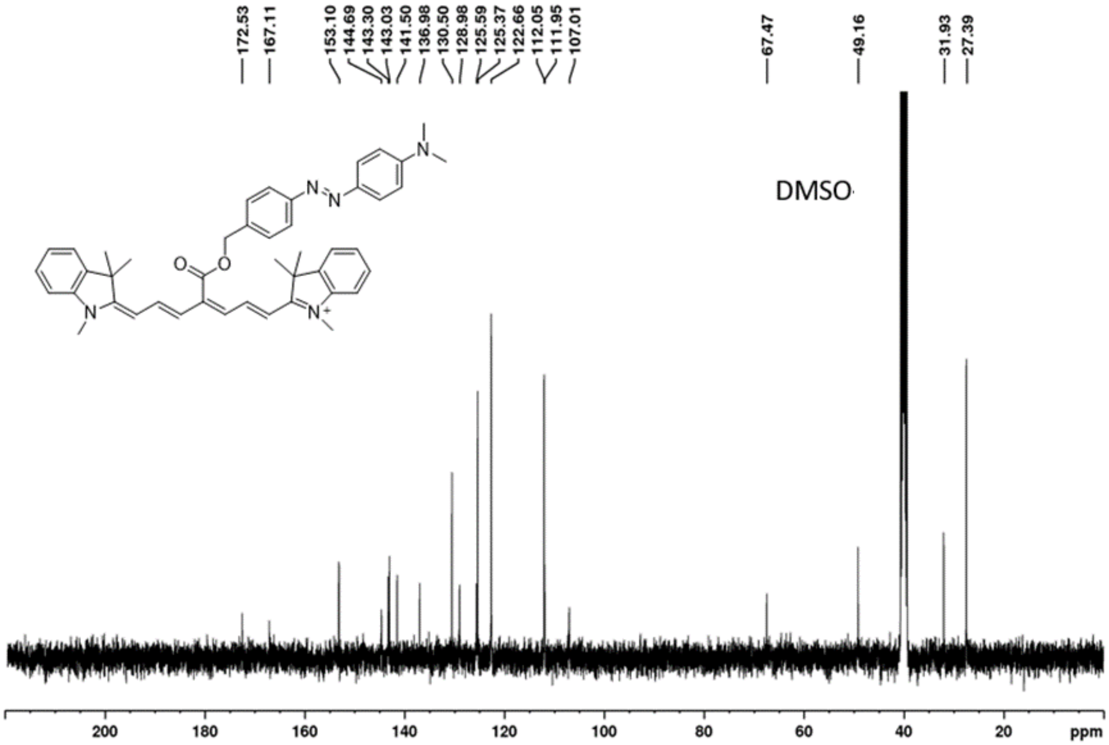

^{13}C NMR spectrum (126 MHz, DMSO-d_6, 25°C) of **aCy7**

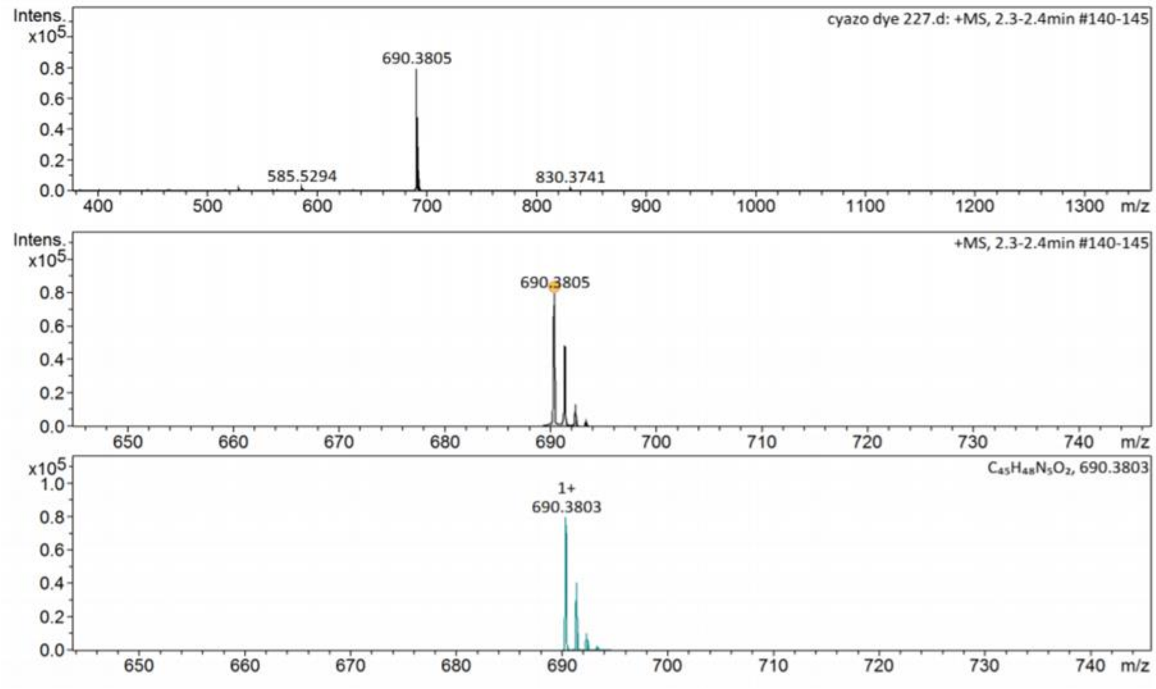

HRMS (ESI-TOF) spectrum of **aCy7**

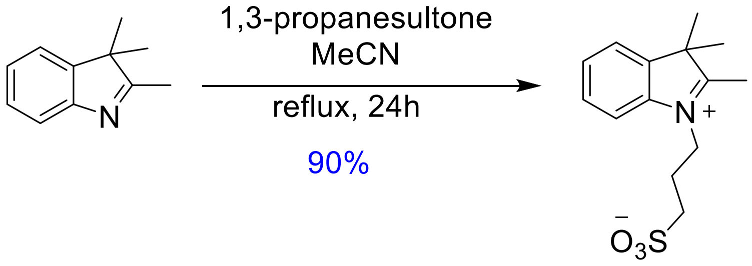

6

Compound **6**. The mixture of 1,3-propanesultone (1.0 mL, 7.5 mmol, 1 eq) and 2,3,3-Trimethyl-3H-indole (2.4 mL, 15 mmol, 2 eq) was refluxed in acetonitrile (20 mL) for 24 hours turning a dark violet. The solution was then cooled to room temperature and a mixture of methanol (20 mL), hexane (100 mL), and diethyl ether (200 mL) was added to the crude followed by sonication for 10 min. The mixture was then vacuum filtered, washed with 50 mL of diethyl ether to afford a solid pink precipitate **6** (1.90 g, 90%). ^{1}H NMR (400 MHz, DMSO-d_6, 25 °C) δ(ppm): 8.05 (d, J = 7.2 Hz, 1H), 7.82 (d, J = 5.9 Hz, 1H), 7.69 – 7.56 (m, 2H), 4.65 (t, J = 8.0 Hz, 2H), 2.83 (s, 3H), 2.62 (t, J = 6.5 Hz, 2H), 2.15 (t, J = 7.8 Hz, 2H), 1.53 (s, 6H).

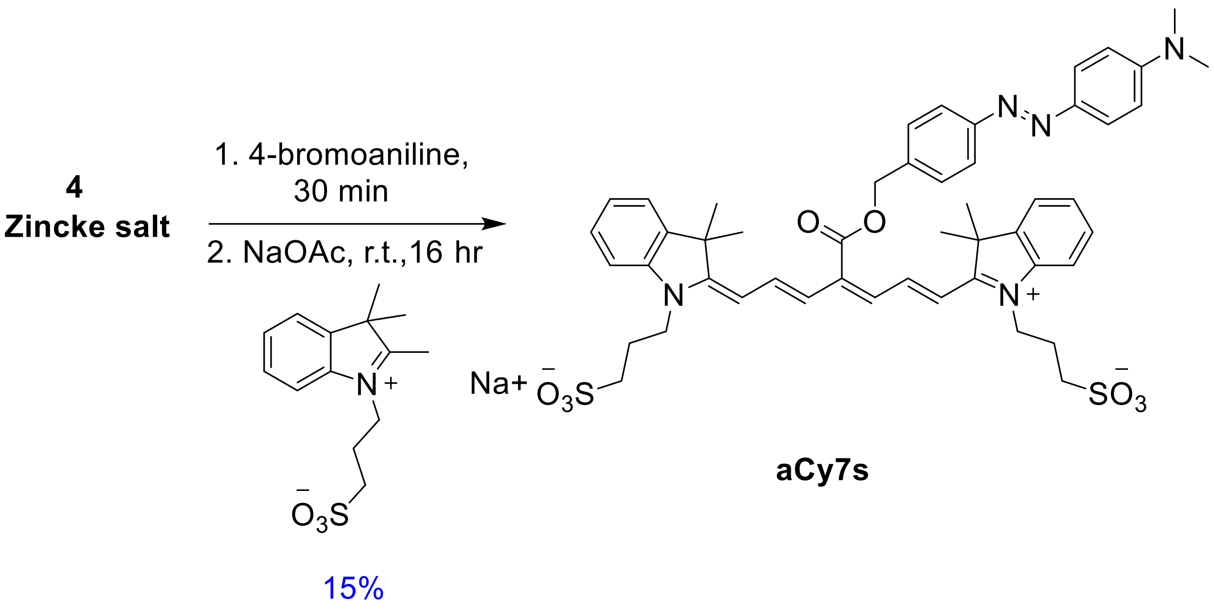

Compound **aCy7s**. A mixture of Zincke salt **4** (200 mg, 0.38 mmol, 1 eq) and 4-bromoaniline (78.26 mg, 0.45 mmol, 1.2 eq) in methanol (10 mL) was stirred for 30 min at room temp, followed by the addition of indole salt **6** (235 mg, 0.83 mmol, 2.2 eq) and sodium acetate (136.84 mg, 1.67 mmol, 4.4 eq). The reaction mixture was stirred overnight at room temperature in the dark, turning a brownish-green color. MeOH was removed under reduced pressure, the residue was suspended in ethyl acetate (10 mL), sonicated, and filtered to remove nonpolar impurities. The filter cake was further purified by reverse phase column chromatography (C18, 50-90% MeOH containing 0.5% TFA in H_2O) to yield **aCy7s** as a green solid (50 mg, 15%).^{1}H NMR (400 MHz, DMSO-d_6, 25°C) δ(ppm): 7.85-7.81 (t, *J* = 8.1 Hz, 4H), 7.80-7.78 (t, *J* = 13.7 Hz, 1H), 7.70 (d, *J* = 8.3 Hz, 2H), 7.52-7.45 (dd, *J* = 8.7 Hz, 4H), 7.41 (t, *J* = 7.7 Hz, 4H), 7.22 (t, *J* = 7.3 Hz, 2H), 6.85 (d, *J* = 8.7 Hz, 2H), 6.68-6.63 (m, *J* = 13.0 Hz, 4H), 5.55 (s, 2H), 4.27 (t, *J* = 7.4 Hz, 4H), 3.08 (s, 6H), 2.55 (t, *J* = 7.4 Hz, 4H), 2.00 (p, *J* = 7.1 Hz, 4H), 1.46 (s, 12 H). ^{13}C NMR (126 MHz, DMSO-d_6, 25°C) δ(ppm): 172.6, 167.0, 153.0, 145.3, 143.0, 141.5, 138.1, 130.4, 128.7, 125.4, 125.2, 123.6, 111.8, 111.7, 107.0, 68.0, 50.2, 48.4, 45.2, 30.2, 26.8, 24.5. HRMS (ESI-TOF) m/z: [M + H]$^+$ calcd for $C_{49}H_{54}N_5O_8S_2^+$ 904.3419, found 904.3415.

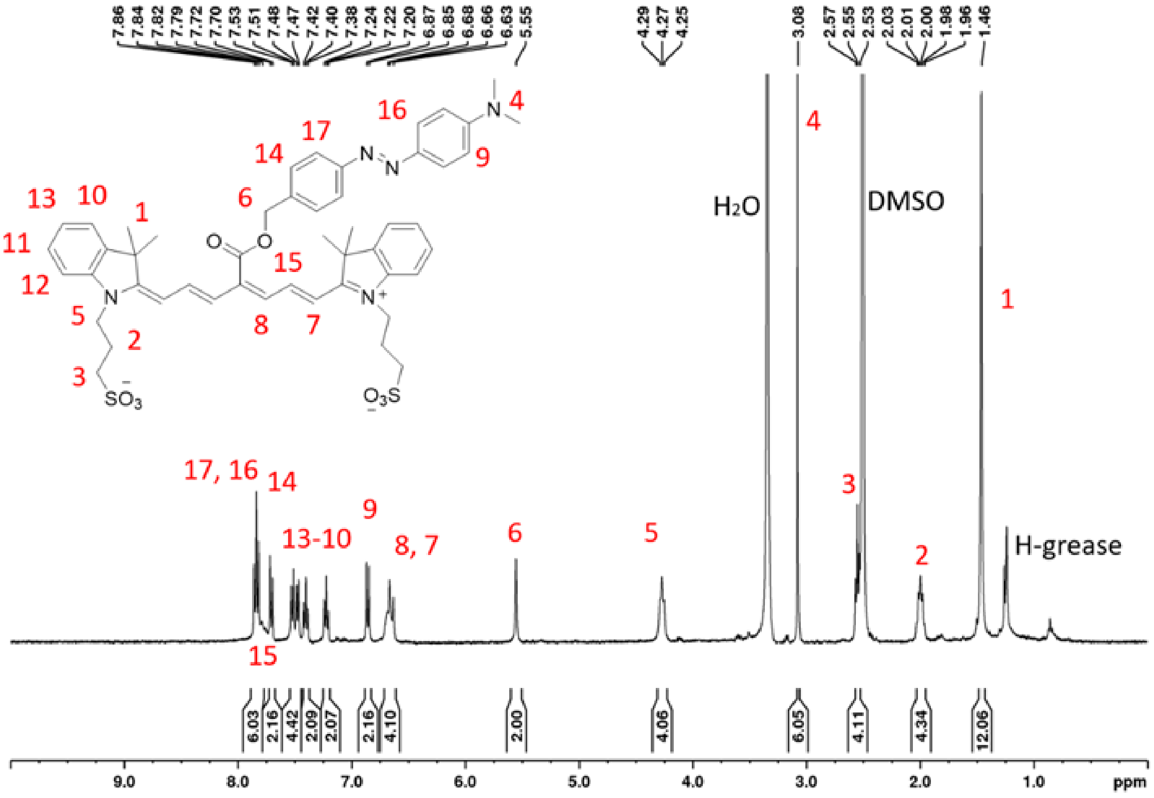

^{1}H NMR spectrum (400 MHz, DMSO-d_6, 25°C) of **aCy7s**

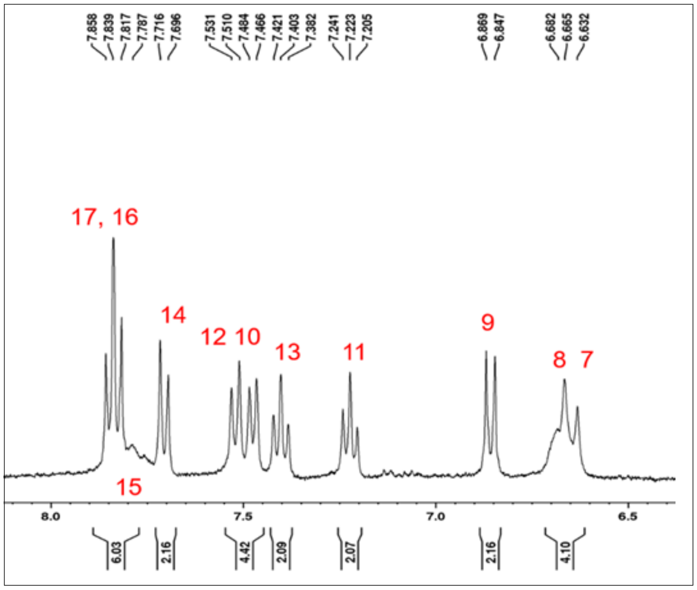

^{1}H NMR spectrum (400 MHz, DMSO-d_6, 25 °C) expanded aromatic region (6.5 - 8.0 ppm) of **aCy7s**

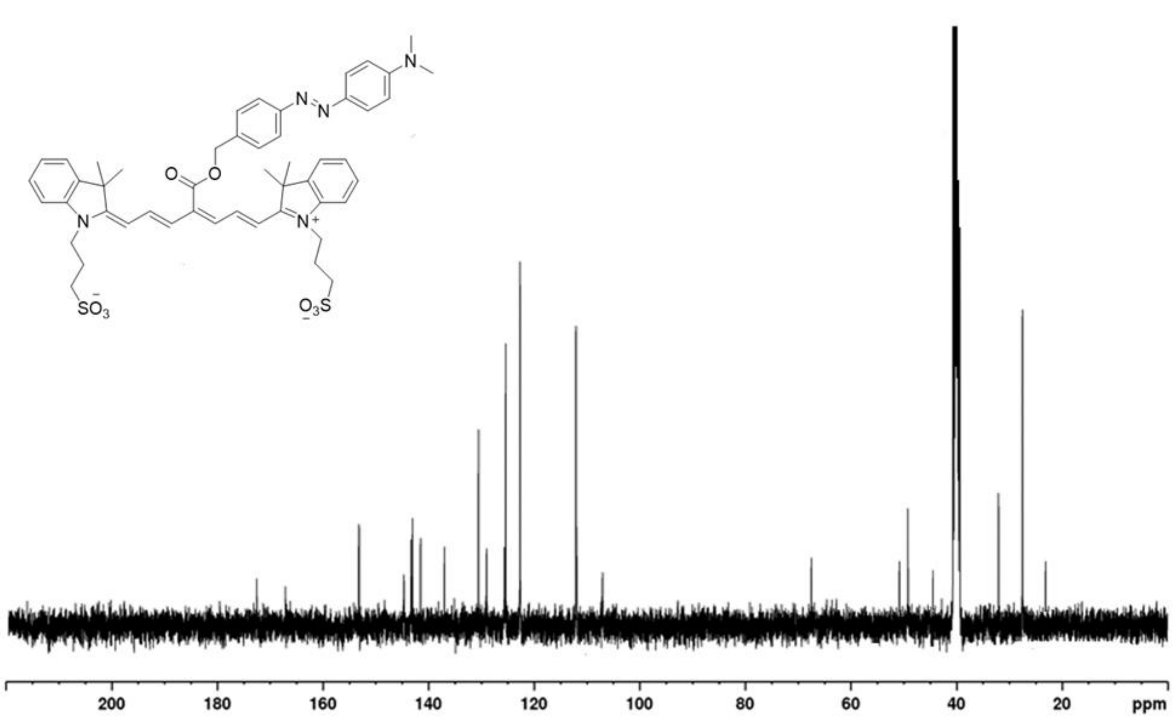

^{13}C NMR spectrum (126 MHz, DMSO-d_6, 25°C) of **aCy7s**

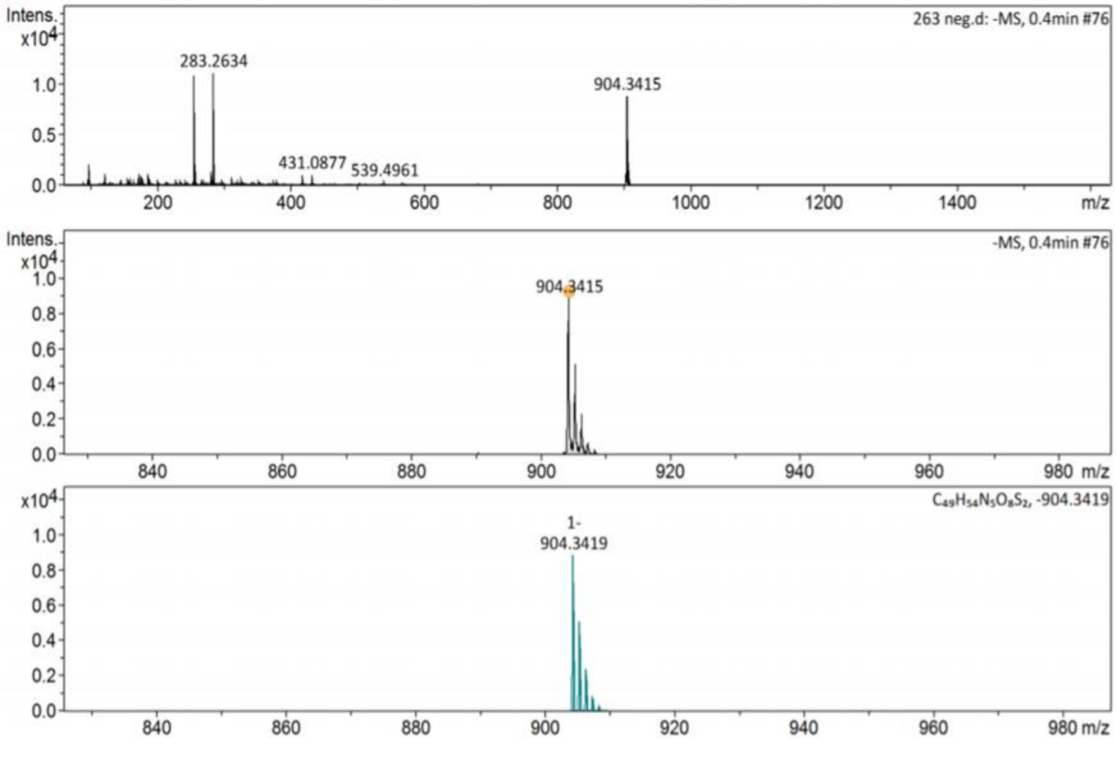

HRMS (ESI-TOF) spectrum of **aCy7s**

10. DFT calculated molecular model of CB7@aCy7

Calculation Method = CAM-B3LYP
Basis Set = 6-31+G(D, P)
Solvation = scrf = (cpcm, solvent = water)
Electronic Energy (Hartree) = -6377.9924
Dipole Moment (Debye) = 23.396042
(No imaginary frequency)

No.	Atom	X	Y	Z
1	C	-2.95285	6.141593	-1.58739
2	H	-3.11878	7.042034	-2.18785
3	C	-2.39116	6.431354	-0.16717
4	H	-2.15873	7.486747	0.007914
5	C	-4.51906	5.471356	0.025767
6	C	-0.89972	5.040798	-1.33211
7	C	-3.51477	6.323312	2.122151
8	H	-3.14794	7.345308	2.261289
9	H	-4.56011	6.26958	2.424924
10	C	-5.21121	5.230894	-2.32853
11	H	-6.16593	5.102767	-1.81921
12	H	-5.26776	6.099895	-2.99377
13	C	-0.20752	5.727232	0.929882
14	H	-0.06909	6.777217	1.208718
15	H	0.728853	5.330037	0.539601
16	C	-1.87427	5.008068	-3.60279
17	H	-2.15731	5.901805	-4.16834
18	H	-0.84407	4.741935	-3.83738
19	C	-1.34893	5.502431	3.207896
20	H	-1.04346	6.52394	3.454271
21	C	-1.10831	4.477293	4.357017
22	H	-0.67453	4.918101	5.259753
23	C	-3.39859	4.564798	3.855656
24	C	0.112293	3.831719	2.471097
25	C	-2.75718	3.077177	5.720759
26	H	-2.22963	3.413859	6.618833
27	H	-3.83221	3.15003	5.882369
28	C	0.54908	2.531412	4.518842
29	H	0.816053	2.96316	5.489379
30	H	1.457318	2.298622	3.963783
31	C	-1.14597	1.10216	5.762003
32	H	-0.75995	1.451957	6.725063
33	C	-1.41535	-0.42683	5.714107
34	H	-1.28959	-0.92822	6.679504

35	C	-3.39869	0.732832	5.240127
36	C	0.304048	0.099416	4.21262
37	C	-3.56696	-1.72472	5.347494
38	H	-3.30978	-2.25817	6.27021
39	H	-4.62028	-1.44667	5.370602
40	C	-0.11611	-2.30874	4.581073
41	H	0.874264	-2.35665	4.129376
42	H	-0.09413	-2.79664	5.561096
43	C	-2.28781	-3.56853	4.152613
44	H	-2.17429	-4.08781	5.110092
45	C	-2.70758	-4.49957	2.981399
46	H	-2.74737	-5.55918	3.254776
47	C	-4.41015	-2.93885	3.37789
48	C	-0.65768	-3.48091	2.47038
49	C	-4.93266	-4.74397	1.768378
50	H	-4.8349	-5.81452	1.977825
51	H	-5.94435	-4.4181	2.008202
52	C	-1.51847	-5.08909	0.808209
53	H	-1.70408	-6.13704	1.067872
54	H	-0.48847	-4.97828	0.471
55	C	-3.73899	-5.2042	-0.44027
56	H	-3.78537	-6.28289	-0.25759
57	C	-4.10645	-4.80014	-1.89702
58	H	-4.29695	-5.65378	-2.55579
59	C	-5.68413	-3.89353	-0.41931
60	C	-1.92015	-4.09585	-1.41974
61	C	-6.151	-3.58218	-2.82558
62	H	-7.13526	-3.37016	-2.40899
63	H	-6.23043	-4.39378	-3.55555
64	C	-2.70944	-3.70692	-3.72389
65	H	-1.63465	-3.5882	-3.85846
66	H	-3.07031	-4.51629	-4.36873
67	C	-4.71455	-2.37912	-4.56022
68	H	-4.93304	-3.16149	-5.29439
69	C	-4.81238	-0.93755	-5.13388
70	H	-5.19046	-0.89617	-6.16061
71	C	-6.30913	-1.16823	-3.344
72	C	-2.59661	-1.37136	-4.50661
73	C	-6.33384	1.005862	-4.49975
74	H	-7.25445	1.065357	-3.91989
75	H	-6.57154	1.074899	-5.5671
76	C	-2.99026	0.747488	-5.72085
77	H	-3.48315	0.835441	-6.69488
78	H	-1.91421	0.654534	-5.86461

79	C	-4.47569	2.679799	-4.97817
80	H	-4.83996	2.791018	-6.00482
81	C	-4.12237	4.028881	-4.29088
82	H	-4.31413	4.906025	-4.91774
83	C	-5.82234	2.96127	-3.07872
84	C	-2.18767	2.714219	-4.46376
85	N	-3.44993	5.995395	0.715465
86	N	-4.21145	5.476553	-1.31535
87	N	-1.18451	5.632789	-0.12298
88	N	-1.93518	5.31365	-2.19327
89	N	-2.43213	3.959418	4.623918
90	N	-2.77678	5.433435	2.989233
91	N	-0.18628	3.525758	3.768446
92	N	-0.54181	4.980887	2.123111
93	N	-2.7964	-0.50511	5.281088
94	N	-2.43955	1.685674	5.495853
95	N	-0.44137	-0.91415	4.765163
96	N	-0.15704	1.292694	4.720551
97	N	-4.02363	-4.0234	2.626716
98	N	-3.37436	-2.61172	4.224705
99	N	-1.67592	-4.27216	1.989287
100	N	-1.01936	-3.03196	3.718998
101	N	-5.307	-4.01174	-1.73579
102	N	-4.73375	-4.51467	0.356628
103	N	-2.93892	-4.06921	-2.34499
104	N	-2.38295	-4.72771	-0.28993
105	N	-5.73853	-0.28285	-4.22949
106	N	-5.70082	-2.38991	-3.50518
107	N	-3.45105	-0.45993	-5.07929
108	N	-3.33493	-2.46831	-4.12339
109	N	-4.97901	4.048081	-3.12369
110	N	-5.51248	2.133348	-4.132
111	N	-2.70829	3.905929	-4.01699
112	N	-3.22098	1.957073	-4.96558
113	O	-5.57414	5.092569	0.517049
114	O	0.113406	4.408937	-1.59877
115	O	-4.60416	4.38131	3.944019
116	O	0.857436	3.184752	1.732932
117	O	-4.58674	0.945595	5.040187
118	O	1.236166	-0.03565	3.431967
119	O	-5.49663	-2.37849	3.326647
120	O	0.398916	-3.24526	1.90022
121	O	-6.70284	-3.3531	-0.01098
122	O	-0.7907	-3.65618	-1.58609

123	O	-7.22097	-0.91695	-2.56712
124	O	-1.38563	-1.24371	-4.38504
125	O	-6.71474	2.779953	-2.26118
126	O	-1.00636	2.39623	-4.44756
127	C	1.604102	0.792846	-0.81057
128	C	1.758626	-0.2085	-1.76758
129	H	0.894514	-0.74298	-2.14076
130	C	3.034067	-0.51305	-2.22318
131	H	3.1577	-1.29929	-2.96133
132	C	4.153473	0.169777	-1.74603
133	C	3.973074	1.187703	-0.80754
134	H	4.831849	1.737208	-0.43462
135	C	2.707838	1.502559	-0.33384
136	H	2.569756	2.28498	0.403693
137	C	-1.91643	0.657301	-0.13884
138	C	-2.32241	1.726078	0.722887
139	H	-1.6121	2.479447	1.039232
140	C	-3.60823	1.829406	1.146254
141	H	-3.89057	2.648742	1.792245
142	C	-4.6064	0.866871	0.748742
143	C	-4.20188	-0.19298	-0.1299
144	H	-4.92381	-0.92801	-0.45635
145	C	-2.91288	-0.27948	-0.55332
146	H	-2.60424	-1.0802	-1.21664
147	C	-6.86411	-0.02596	0.789706
148	H	-6.97599	-0.06115	-0.2968
149	H	-7.81722	0.255929	1.230126
150	H	-6.58951	-1.02018	1.151064
151	C	-6.25958	2.047562	2.088776
152	H	-5.71431	1.980882	3.033402
153	H	-7.32328	1.94893	2.290527
154	H	-6.07836	3.022887	1.630902
155	N	0.332767	1.117918	-0.2862
156	H	0.300718	1.896425	0.387778
157	N	-0.69206	0.415723	-0.60516
158	N	-5.85872	0.965991	1.182097
159	C	5.527978	-0.1968	-2.21628
160	O	6.185251	-0.9158	-1.13309
161	H	6.134272	0.682001	-2.44568
162	H	5.498645	-0.8438	-3.0939
163	C	7.424018	-1.34063	-1.37247
164	O	7.995395	-1.19385	-2.43342
165	C	8.05113	-1.99854	-0.17148
166	C	9.240924	-1.45531	0.329323

167	C	9.882306	-0.32241	-0.15885
168	C	11.05885	0.18739	0.38949
169	C	11.73615	1.315282	-0.06391
170	C	11.38237	2.2536	-1.22226
171	C	12.52543	3.244852	-1.15546
172	N	12.87258	1.742605	0.520352
173	C	13.37622	2.900618	-0.10983
174	C	12.80154	4.35307	-1.93644
175	C	13.9421	5.109584	-1.65242
176	C	14.78348	4.750754	-0.60081
177	C	14.51376	3.634383	0.192561
178	H	12.1487	4.635474	-2.75656
179	H	14.17381	5.981536	-2.25449
180	H	15.66568	5.346077	-0.39044
181	H	15.17784	3.368543	1.005958
182	C	7.4724	-3.13347	0.403223
183	C	6.310965	-3.7728	-0.02243
184	H	8.0015	-3.56286	1.250724
185	C	5.817048	-4.92197	0.592207
186	H	5.773449	-3.34747	-0.86139
187	C	4.663115	-5.61254	0.228337
188	H	6.3869	-5.30918	1.430008
189	C	3.666661	-5.30704	-0.89259
190	N	4.264601	-6.71791	0.886122
191	C	2.677795	-6.44089	-0.72308
192	C	3.073144	-7.24429	0.341462
193	C	1.529249	-6.74624	-1.43161
194	C	0.785622	-7.8685	-1.05623
195	C	1.196225	-8.66303	0.012922
196	C	2.352951	-8.3629	0.734326
197	H	1.203334	-6.1262	-2.2607
198	H	-0.11773	-8.12243	-1.60048
199	H	0.609863	-9.53144	0.294037
200	H	2.658538	-8.99036	1.562761
201	C	10.04022	2.971679	-0.98119
202	H	10.03605	3.481101	-0.01455
203	H	9.198494	2.278174	-1.00873
204	H	9.886656	3.719622	-1.76285
205	C	11.38841	1.520222	-2.57745
206	H	12.33365	0.995293	-2.73506
207	H	11.26437	2.250061	-3.38132
208	H	10.57549	0.796394	-2.65292
209	C	2.965971	-3.95313	-0.67073
210	H	2.495865	-3.90633	0.314517

211	H	2.182659	-3.8261	-1.42199
212	H	3.661785	-3.11828	-0.76352
213	C	4.328408	-5.37617	-2.28232
214	H	5.051252	-4.57222	-2.42896
215	H	3.558316	-5.28003	-3.05163
216	H	4.840006	-6.33081	-2.42722
217	C	4.970332	-7.29863	2.016785
218	H	5.027402	-6.58017	2.837475
219	H	5.979495	-7.59534	1.722418
220	H	4.43533	-8.17899	2.36137
221	C	13.50298	1.091101	1.656511
222	H	12.82294	1.0775	2.511255
223	H	14.39961	1.638111	1.934049
224	H	13.7832	0.067086	1.399292
225	H	9.680637	-1.96146	1.185198
226	H	9.442488	0.176332	-1.0134
227	H	11.4769	-0.34282	1.238372

References

(1) Peck, E. M.; Liu, W.; Spence, G. T.; Shaw, S. K.; Davis, A. P.; Destecroix, H.; Smith, B. D. Rapid Macrocycle Threading by a Fluorescent Dye-Polymer Conjugate in Water with Nanomolar Affinity. *J. Am. Chem. Soc.* **2015**, *27*, 8668–8671, https://doi.org/10.1021/jacs.5b03573.

(2) Dempsey, J. M.; Zhai, C.; McGarraugh, H. H.; Schreiber, C. L.; Stoffel, S. E.; Johnson, A.; Smith, B. D. High Affinity Threading of a New Tetralactam Macrocycle in Water by Fluorescent Deep-Red and near-Infrared Squaraine Dyes. *Chem. Commun.* **2019**, *85*, 12793–12796. https://doi.org/10.1039/c9cc05244e.

(3) Bindfit software: http://app.supramolecular.org/bindfit/

(4) Zhou, Y.; Maiti, M.; Sharma, A.; Won, M.; Yu, L.; Miao, L. X.; Shin, J.; Podder, A.; Bobba, K. N.; Han, J.; Bhuniya, S.; Kim, J. S. Azo-Based Small Molecular Hypoxia Responsive Theranostic for Tumor-Specific Imaging and Therapy. *J. Control. Release.* **2018**, *288*, 14–22. https://doi.org/10.1016/j.jconrel.2018.08.036.

(5) Štacková, L.; Štacko, P.; Klán, P. Approach to a Substituted Heptamethine Cyanine Chain by the Ring Opening of Zincke Salts. *J. Am. Chem. Soc.* **2019**, *17*, 7155–7162. https://doi.org/10.1021/jacs.9b02537.
